# CryoEM structures of Arabidopsis DDR complexes involved in RNA-directed DNA methylation

**DOI:** 10.1038/s41467-019-11759-9

**Published:** 2019-09-02

**Authors:** Somsakul Pop Wongpalee, Shiheng Liu, Javier Gallego-Bartolomé, Alexander Leitner, Ruedi Aebersold, Wanlu Liu, Linda Yen, Maria A. Nohales, Peggy Hsuanyu Kuo, Ajay A. Vashisht, James A. Wohlschlegel, Suhua Feng, Steve A. Kay, Z. Hong Zhou, Steven E. Jacobsen

**Affiliations:** 1 Department of Molecular, Cellular and Developmental Biology, University of California, Los Angeles (UCLA), Los Angeles, CA 90095 USA; 2 Department of Microbiology, Faculty of Medicine, Chiang Mai University, Chiang Mai, 50200 Thailand; 3 Department of Microbiology, Immunology and Molecular Genetics, UCLA, Los Angeles, CA 90095 USA; 4 California NanoSystems Institute (CNSI), UCLA, Los Angeles, CA 90095 USA; 5 Department of Biology, Institute of Molecular Systems Biology, ETH Zürich, 8093 Zürich, Switzerland; 6 Faculty of Science, University of Zürich, 8057 Zürich, Switzerland; 7 Zhejiang University-University of Edinburgh Institute, Zhejiang University School of Medicine, 310058 Hangzhou, P. R. China; 8 Keck School of Medicine, University of Southern California, Los Angeles, CA 90089 USA; 9 Department of Biological Chemistry, UCLA, Los Angeles, CA 90095 USA; 10 Howard Hughes Medical Institute (HHMI), UCLA, Los Angeles, CA 90095 USA

**Keywords:** Cryoelectron microscopy, DNA methylation, Plant molecular biology, Cryoelectron microscopy

## Abstract

Transcription by RNA polymerase V (Pol V) in plants is required for RNA-directed DNA methylation, leading to transcriptional gene silencing. Global chromatin association of Pol V requires components of the DDR complex DRD1, DMS3 and RDM1, but the assembly process of this complex and the underlying mechanism for Pol V recruitment remain unknown. Here we show that all DDR complex components co-localize with Pol V, and we report the cryoEM structures of two complexes associated with Pol V recruitment—DR (DMS3-RDM1) and DDR′ (DMS3-RDM1-DRD1 peptide), at 3.6 Å and 3.5 Å resolution, respectively. RDM1 dimerization at the center frames the assembly of the entire complex and mediates interactions between DMS3 and DRD1 with a stoichiometry of 1 DRD1:4 DMS3:2 RDM1. DRD1 binding to the DR complex induces a drastic movement of a DMS3 coiled-coil helix bundle. We hypothesize that both complexes are functional intermediates that mediate Pol V recruitment.

## Abstract (web summary)

RNA polymerase V transcription in plants, which is needed DNA methylation and transcriptional silencing, requires components of the DDR complex. Here the authors show that all components of the DDR complex co-localize with Pol V and report the cryoEM structures of two complexes associated with Pol V recruitment.

## Introduction

Epigenetic regulation through DNA methylation prevents gene transcription and transposon movement in many eukaryotes. In plants, de novo DNA methylation is guided by small RNAs that target specific genomic regions in a process called RNA-directed DNA methylation (RdDM)^1^. RdDM requires two plant-specific RNA polymerases known as RNA polymerase IV (Pol IV) and RNA polymerase V (Pol V), and a number of accessory proteins with different functions^2,3^.

The canonical RdDM pathway involves several steps that can be divided in two major arms. Briefly, in the first arm of the pathway Pol IV transcripts are copied into double-stranded RNAs (dsRNAs) by RNA-DEPENDENT RNA POLYMERASE 2 (RDR2) and diced by different DICER-LIKE proteins into small interfering RNAs (siRNAs), which are then loaded into ARGONAUTE 4 (AGO4) protein^4^. In the second arm of the pathway, transcribing Pol V recruits siRNA-loaded AGO4, which in turn triggers the recruitment of the DNA methyltransferase DOMAINS REARRANGED METHYLTRANSFERASE 2 (DRM2), to mediate de novo methylation of cytosines in all sequence contexts (CG, CHG, and CHH, where H represents A, C, or T). Since Pol V occupancy dictates RdDM loci^5^, it is crucial to determine how Pol V is targeted to chromatin. Two methyl-DNA binding SU(VAR)3–9 homologs—SUVH2 and SUVH9—as well as components of the DDR complex—DEFECTIVE IN RNA-DIRECTED DNA METHYLATION 1 (DRD1), a putative SNF2-containing chromatin remodeling protein;^6^ DEFECTIVE IN MERISTEM SILENCING 3 (DMS3), a structural maintenance of chromosomes (SMC) hinge domain-containing protein^7^; and RNA-DIRECTED DNA METHYLATION 1 (RDM1), a plant-specific protein with a conserved DUF1950 domain^4,8^—have been shown to be required for global chromatin association of Pol V and therefore exhibit a strong RdDM mutant phenotype^5,9^. Previous studies have shown physical interactions among DDR proteins^10,11^, between SUVH2/SUVH9 and DMS3^12^, and between DRD1 and Pol V^11^. Artificial tethering of SUVH9, DMS3, or RDM1 to the *FWA* locus in different RdDM mutant backgrounds has demonstrated genetically that the DDR complex acts downstream of SUVH2/SUVH9^13^. All together, these observations have positioned the DDR complex as a key component that acts downstream of the methylation readers SUVH2/SUVH9 to recruit Pol V to RdDM loci. However, the assembly characteristics of the DDR complex and the underlying mechanism of Pol V recruitment are unknown.

Here we report cryoEM structures of the DR (DMS3-RDM1) and DDR′ (DRD1 peptide-DMS3-RDM1) complexes. Our structures reveal that DRD1 binds to a complex centralized by an RDM1 dimer core with two dimers of DMS3 bound on opposite sides. A striking conformational change occurs as a flexible coiled-coil (CC) domain of DMS3 in the DR complex becomes stabilized by binding of DRD1 in the DDR′ complex. We propose that the binding of DRD1 to the DR complex might represent an assembly step of DDR complex formation that possibly leads to the recruitment of RNA Pol V.

## Results

### DMS3 and RDM1 form a stable complex in vivo and in vitro

**Fig. 1:**
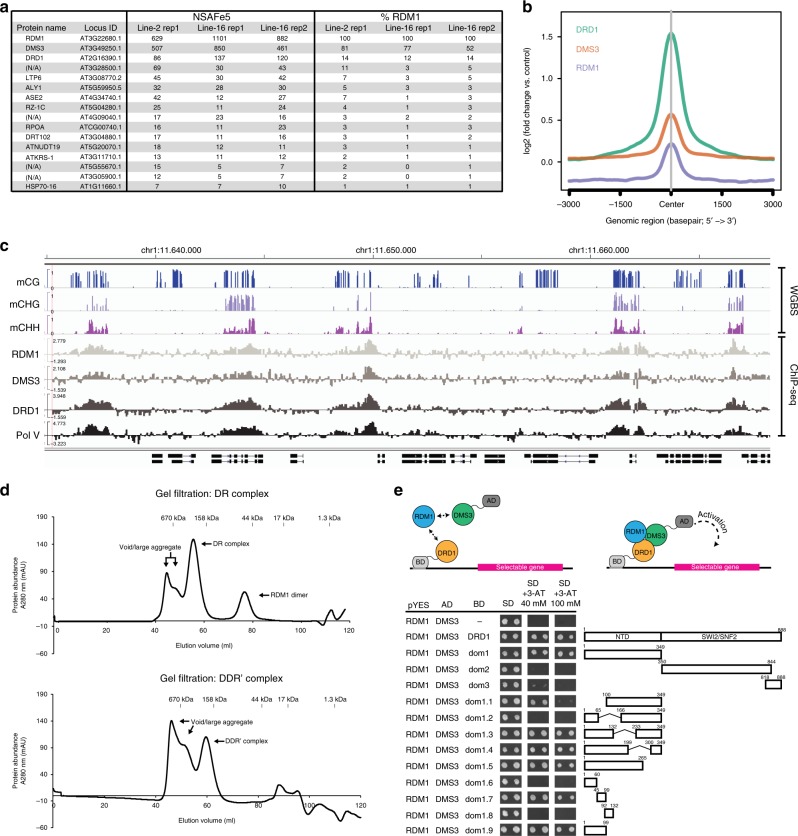
DMS3, RDM1, and DRD1 form complexes in vivo and in vitro. **a** List of proteins co-purified with RDM1 identified by mass spectrometry (MS). Only proteins present in all three independent IP experiments using RDM1-3xFLAG lines but absent in two independent IP experiments using untransformed WS control are shown. Normalized spectral abundance factor value (NSAF × 10^5^)^59^ is indicated for each protein. Estimated stoichiometry is shown as the percentage of RDM1 using NSAF values. All proteins except for the RDM1, DMS3 and DRD1 are common contaminants present in different IP-MS experiments performed in our laboratory. N/A is denoted when protein name not available. **b** Metaplot showing DRD1, DMS3 and RDM1 ChIP-seq signals over Pol V peaks. **c** Representative screenshot of a genomic region showing CG, CHG and CHH methylation levels from WGBS in wild-type control^31^, as well as ChIP-seq signals of RDM1, DMS3, DRD1 and Pol V,  expressed as log_2_ ratio over controls. **d** Gel filtration analyses of His tag affinity-purified DR complex (top) or Strep II tag affinity-purified DDR′ complex (bottom) showing that both complexes are stable and isolatable from *E. coli*. Protein molecular weight marker is indicated above each profile. Peaks eluted around 670 kDa represent void/large aggregate and will not be further characterized. **e** Y3H experiment showing interactions of the DMS3-RDM1 complex with different DRD1 fragments. Upper panel: explanatory cartoon of Y3H technique. BD: GAL4 binding domain fusion, AD: GAL4 activation domain fusion. pYES: expression plasmid under a constitutive ADH1 promoter. Growth of two independent colonies in minimal medium (SD) supplemented with increasing concentrations of 3-amino-1,2,4-triazole (3-AT) is shown. Cartoon depicting the length of the different DRD1 fragments tested is shown on the right. Numbers above each fragment indicate the position of the first and last amino acid with respect to full-length DRD1. N-terminal domain (NTD) and SWI2/SNF2 domain are indicated

The DDR complex was first proposed by Law et al. as a complex of three co-precipitated proteins found in immunoprecipitation followed by mass spectrometry (IP-MS) experiments designed to identify interactors of DMS3 and DRD1^11^. We performed a reciprocal IP-MS experiment with lines expressing RDM1-3xFLAG (Fig. 1a and Supplementary Fig. 1a). As expected, we detected all three components of the DDR complex as the most abundant proteins in these samples. Importantly, the rest of the proteins identified in the experiment represent common contaminants obtained in other independent IP-MS experiments in our laboratory. Interestingly, we observed that RDM1 is more efficient at co-precipitating DMS3 compared to DRD1. Consistent with this observation, DMS3-3xFLAG IP-MS results showed higher abundance of RDM1 compared to DRD1^11^. We performed ChIP-seq experiments to analyze the genome localization of the different DDR components. The results showed strong overlap in the localization between each of the DDR proteins over Pol V sites, suggesting that they function together in vivo (Fig. 1b, c and Supplementary Fig. 1b).

To gain more insight into how the DDR components interact with each other, we carried out yeast 2-hybrid experiments (Y2H). Our data confirmed previous observations that DMS3 and RDM1 can homo- and heterodimerize^10^. However, we did not detect interactions between DRD1 and DMS3 or RDM1, or DRD1 with itself (Supplementary Fig. 1c). In an attempt to detect these interactions in a different system, we co-expressed the DDR proteins in *E. coli* and found that DMS3 and RDM1 were well expressed and formed a large complex of approximate 200–300 kDa by themselves (Fig. 1d, top), even in the absence of DRD1, which had poor solubility (Supplementary Fig. 1d). These results, together with the results obtained in IP-MS experiments, support the idea that, in addition to their interaction with DRD1, DMS3 and RDM1 form a separate stable complex, which we named the DR complex.

### Overall structure of the DR complex

**Fig. 2:**
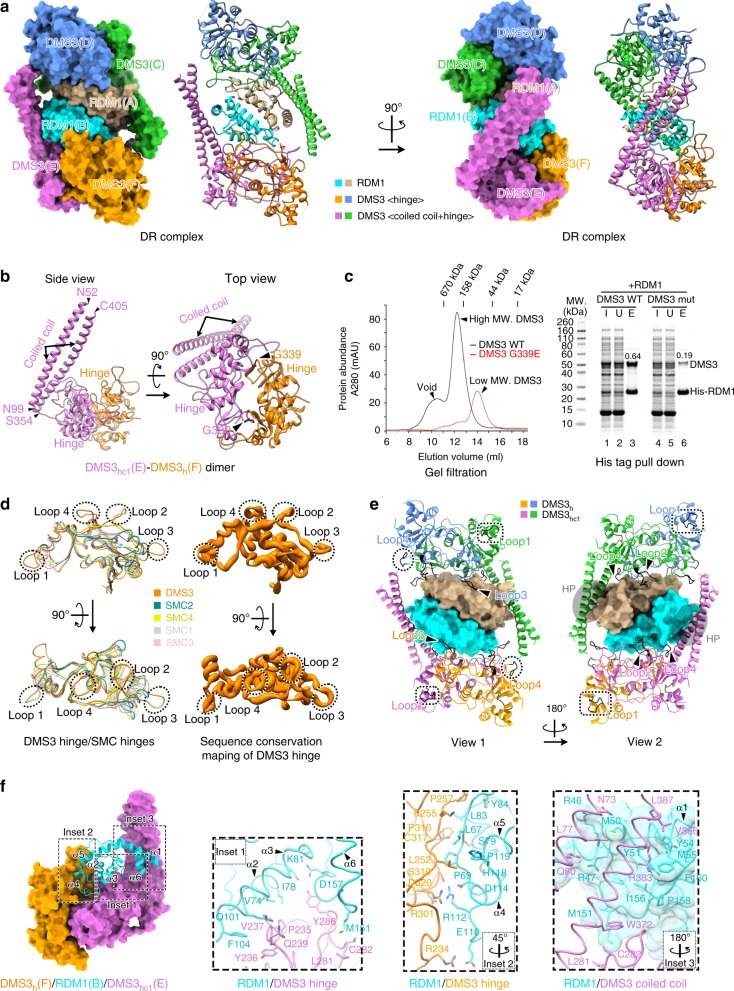
Structure of the DR (DMS3-RDM1) complex. **a** Two orthogonal views of the overall DR complex structure. Left panel of each view: surface representation; Right panel of each view: ribbon representation sharing the same view as the corresponding left panel. Chain names are shown inside the parentheses. **b** Two orthogonal views of the DMS3 dimer (DMS3_hc1_-DMS3_h_) in the DR complex. Chain names are shown inside the parentheses. **c** Left: a gel filtration profile of His tag affinity-purified DMS3 WT and DMS3 G339E. Protein molecular weight marker is indicated above. Right: a representative gel from His tag pulldown experiment. His tag-RDM1 is co-expressed with either DMS3 WT or DMS3 G339E in *E. coli*. Lysates are used in the pulldown experiment on magnetic Ni-NTA agarose bead. Input (I), unbound (U), and eluate (E) are analyzed by SDS-PAGE and protein staining. Numbers on eluted DMS3 bands represent normalized DMS3/RDM1 signals (see Methods section). The uncropped image is shown in Supplementary Fig. 11a. **d** Left: structural comparison of the hinge domains of DMS3 and those of SMC1-4. Four unique loops of the DMS3 hinge are identified as loop 1 to loop 4 (from N- to C-terminal). PDB IDs of SMC1 and 3, and SMC2 and 4 used in the analysis are 2WD5 and 4RSI, respectively. Right: mapping sequence conservation onto the DMS3 hinge domain structure sharing the same view as the corresponding left panel. The plant species used for DMS3 sequence conservation analysis are the same as the species in Supplementary Fig. 6b. Thicker ribbon regions represent more conserved sequences. **e** Interactions of DMS3 hinge’s loops in the DR complex. Hydrophobic pockets (HP) are marked with translucent gray ellipses. **f** Extensive interactions between DMS3 and RDM1. Chain names are shown inside the parentheses. The inset views are the interactions between RDM1 and different DMS3 domains. Interaction between the HP of RDM1 and DMS3 CC is shown in inset 3

Using single-particle cryoEM, we determined the structure of the DR complex to an overall resolution of 3.6 Å (Supplementary Fig. 2–5 and Table 1). The cryoEM structure shows that the stoichiometry of DMS3:RDM1 is 4:2 (Fig. 2a). The two RDM1 molecules in the DR complex form a dimer at the center of the complex (Fig. 2a). RDM1 dimerization is mainly mediated by hydrophobic interactions, which is consistent with the crystal structure of RDM1^8^. Surrounding this RDM1 dimer core are four DMS3 molecules that form two dimers, each binding to the exposed sides of a RDM1 monomer of the dimer core, opposing one another. In addition, the two DMS3 dimers bridge to each other across the RDM1 dimer core using two CC arms (Fig. 2a). Interestingly, while the two DMS3 dimers are similar to one another, the two monomers within each DMS3 dimer differ in their structures: one shows both visible hinge and CC domains, while the other monomer has a visible hinge but unresolved CC domain in the structure (Fig. 2b). For clarity, they are referred to as DMS3_hc1_ and DMS3_h_, respectively.

**Table 1:** CryoEM data collection, refinement and validation statistics

	DR complex (EMD-20080) (PDB 6OIS)	DDR′ complex (EMD-20081) (PDB 6OIT)
*Data collection and processing*		
Magnification	130,000	130,000
Voltage (kV)	300	300
Electron exposure (e^–^/Å^2^)	52.4	47.2
Defocus range (μm)	−1.5 to −2.5	−1.5 to −2.5
Pixel size (Å)	1.07	1.07
Symmetry imposed	C1	C1
Initial particle images (no.)	1,543,489	2,482,628
Final particle images (no.)	314,414	620,248
Map resolution (Å)	3.6	3.5
FSC threshold	0.143	0.143
Map resolution range (Å)	3.0–5.0	3.0–5.0
*Refinement*		
Initial model used	n/a	n/a
Model resolution (Å)	4.6	4.0
FSC threshold	0.5	0.5
Model resolution range (Å)	4.6	4.0
Map sharpening *B* factor (Å^2^)	−138.9	−194.1
Model composition		
Non-hydrogen atoms	10,732	12,085
Protein residues	1365	1536
Ligands	–	–
*B* factors (Å^2^)		
Protein	75.6	30.6
Ligand	–	–
R.m.s. deviations		
Bond lengths (Å)	0.01	0.01
Bond angles (°)	1.27	1.25
Validation		
MolProbity score	1.62	1.55
Clashscore	5.99	5.33
Poor rotamers (%)	0.59	0.53
Ramachandran plot		
Favored (%)	95.82	96.15
Allowed (%)	4.18	3.85
Disallowed (%)	0.00	0.00

### DMS3 and its extensive interactions with RDM1

Compared to bona fide SMC proteins, DMS3 lacks the ABC-type ATPase head but contains a SMC hinge domain and has a much shorter flanking CC domain (Supplementary Fig. 6a). As predicted, DMS3 contains a central SMC hinge domain flanked by two ~50 residues long helices (Fig. 2b). N-terminal region aa. 1–42 and C-terminal region aa. 410–420 were not visible in all DMS3 monomers of our structures, probably due to flexibility. DMS3 dimerizes using two hinge domains in a hand-shake configuration, forming a donut shape (Fig. 2b). Dimerization is mostly mediated by two reciprocal contacts between two β strands (residues 225–229 and residues 334–338) from each monomer, which fold into a continuous β-sheet system, spanning across the two monomers (Supplementary Fig. 8). Each DMS3 folds 180° around its hinge domain, allowing the alpha helices on the N- and C-termini to form an antiparallel CC arm (Fig. 2b). This type of dimerization and folding is consistent with that of SMC family members^14–17^. Interestingly, an ethyl methanesulfonate (EMS) mutagenesis screening in Arabidopsis identified a G339E mutation of DMS3 that resulted in loss of de novo DNA methylation^7^. Our cryoEM structure shows that this G339 residue is located at the dimerization interface between the two hinge domains (Fig. 2b and Supplementary Fig. 8). Mutation from a no-side chain glycine to a bulky glutamate would create steric clashes between the two monomers that could disrupt this dimerization interface (Supplementary Fig. 8). Consistent with this prediction, we found that the size distribution of recombinant DMS3 WT and G339E mutant were drastically different from each other in gel filtration profiles—with the G339E mutant showing elution at a smaller molecular weight (Fig. 2c, left).

When we compared the structure of DMS3 hinge with those of SMC1–4, we found that while most regions of the hinge domains are highly similar, DMS3 possesses four distinct loops—i.e., loops 1–4 (Fig. 2d). Furthermore, sequence alignment of the DMS3 orthologs from different plant species indicated that the four loops are conserved among DMS3 orthologs (Fig. 2d, right and Supplementary Fig. 6b). Loop 1 is located most distal to the core, contributing to DMS3 dimerization; loops 2 and 3 face toward the center of the DR complex, participating in the DMS3-RDM1 interactions; loop 4 is on lateral side, contacting either RDM1 or the CC domain from the other DMS3 dimer (Fig. 2e). It is worth noting that DMS3 dimer interacts with RDM1 in an asymmetric way, causing the same loops from the two monomers to participate in different interactions. Some of these loops are not interacting with other residues in the complex and their functions are thus unknown.

Our cryoEM structure of the DR complex also reveals numerous interactions between DMS3 dimer and RDM1, with a buried interface with an estimated area of ~31% of the RDM1 dimer surface area. Both the hinge dimer and the CC arm of DMS3 interact with RDM1 (Fig. 2f). The hinge dimer-RDM1 interaction is mainly contributed by hydrophobic residues, while few residues are involved in hydrogen bond networks (Fig. 2f, inset 1 and 2). Importantly, the G339E mutation, which disrupted the DMS3 dimer, resulted in a severe loss of DMS3-RDM1 interaction in a pulldown experiment (Fig. 2c, right). This indicates that DMS3 dimerization is crucial for stable interaction between DMS3 and RDM1 in the DR complex. In addition, RDM1 contains a hydrophobic pocket (HP) formed by helix α1, α6, and the last C-terminal segment of RDM1 (Fig. 2f). This pocket binds to CHAPS in a crystal structure^8^, and a mutation (Y51A + Y54A) designed to disrupt this pocket failed to complement an *rdm1* mutant^10^. Notably, both pockets are occupied by the CC arm of DMS3 in our cryoEM structure (Fig. 2e, f, inset 3). Together, our observations show that RDM1 dimerization at the center of the complex brings together two DMS3 dimers to form a 4 DMS3:2 RDM1 stoichiometry (Fig. 2a); this dimerization seems to play a critical role in maintaining the intact architecture of the complex (also see the Discussion section).

### DRD1 binding in the DDR′ complex induces a drastic CC movement

**Fig. 3:**
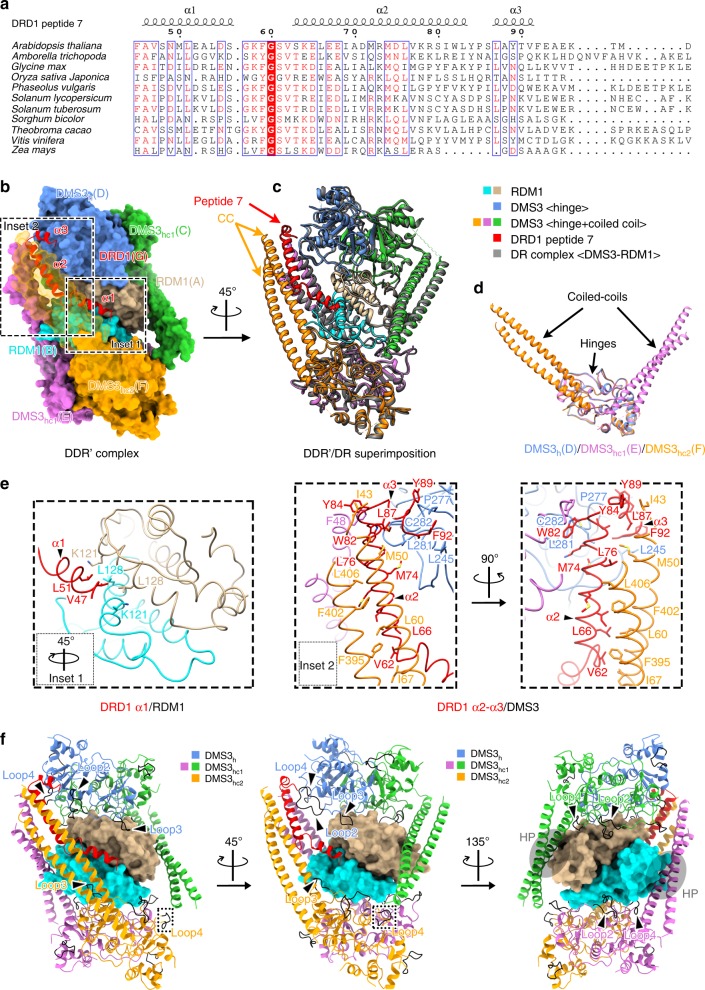
Binding of DRD1 to the DR complex induces a drastic coiled-coil movement. **a** Sequence comparison of DRD1 peptide 7 from multiple plant species using T-Coffee server^60^. Red background marks sequences with strict identity (100%); blue box marks sequences with more than 60% similarity; red text marks conserved amino acids. **b** Overall structure of the DDR′ complex (DRD1 peptide 7-DMS3-RDM1). Chain names are shown inside the parentheses. DMS3 and RDM1 are shown as surface (the CC domain of DMS3_hc2_(F) in front of DRD1 peptide 7 is shown transparently), while DRD1 peptide 7 is shown as ribbon. **c** Superimposition of the DR (all in gray) and DDR′ complexes revealing structural differences. **d** Structural comparison between DMS3_h_, DMS3_hc1_, and DMS3_hc2_. Chain names are shown inside the parentheses. **e** The interactions between DRD1 peptide 7 and DMS3/RDM1. All insets are taken from Fig. 3b. Inset 1: residual interactions between DRD1 α1 segment and a cleft at the RDM1 dimer interface. Inset 2: two orthogonal views showing the residual interactions between DRD1 α2–α3 segments and DMS3. **f** Loop 2 to loop 4 in the DDR′ complex contribute to the interaction of DMS3 with DRD1 and RDM1. HP, hydrophobic pocket of RDM1

DRD1 is a 100-kDa member of the SNF2 protein family that possesses a helicase-like SWI2/SNF2 domain on its C-terminus. Except for a small conserved region (aa. 238–302) preceding the SWI2/SNF2 domain, most of the N-terminal domain (NTD) is not conserved in other Arabidopsis SWI2/SNF2-containing proteins (Supplementary Fig. 9). This uncharacterized domain could thus potentially mediate the specific interaction of DRD1 with the DR complex. Even though DRD1 is a part of the DDR complex, our Y2H experiment failed to show any binary interactions with DMS3 or RDM1 (Supplementary Fig. 1c). However, we found that when all three proteins were expressed together in a yeast 3-hydrid system (Y3H), DRD1 interaction was detected (Fig. 1e). These data suggest that DMS3-RDM1 interaction is a prerequisite for a stable interaction of DRD1. Due to poor solubility of the full-length DRD1 in *E. coli* (Supplementary Fig. 1d), we utilized the Y3H system to search for a soluble minimal region of DRD1 capable of interacting with the other proteins (Fig. 1e). We identified a DRD1 region—which we called peptide 7—that was sufficient and required for interaction of DRD1 with DMS3 and RDM1 (Fig. 1e, dom1.7). Sequence alignment showed that peptide 7 is conserved among the DRD1 proteins from different plant species but not found in other SWI2/SNF2 proteins (Fig. 3a and Supplementary Fig. 9). When co-expressed in *E. coli*, DMS3, RDM1, and DRD1 peptide 7 formed a stable and isolatable complex that we called the DDR′ complex (Fig. 1d). Using single-particle cryoEM, we determined the structure of the DDR′ complex to an overall resolution of 3.5 Å. While the overall structural framework of the DDR′ complex is similar to that of the DR complex, DDR′ exhibits two significant structural differences (Fig. 3b–d).

**Fig. 4:**
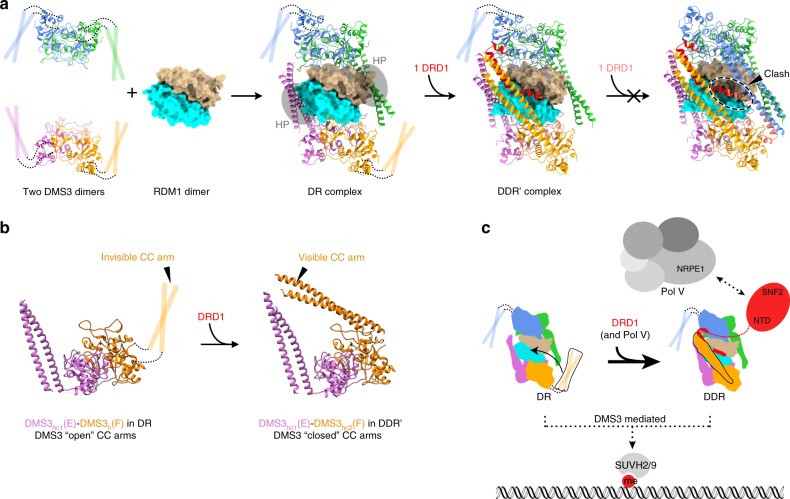
Possible assembly pathway of the DDR complex that leads to Pol V recruitment. **a** The assembly characteristics of the DR and DDR′ complex. The DR complex is a prerequisite for binding of DRD1 peptide 7 but can accommodate only a single DRD1 peptide. Steric clash between the two peptides is expected if another DRD1 peptide binds to the complex. HP, hydrophobic pocket of RDM1. **b** DRD1 binding converts the DMS3 dimer from “open” to “closed” state. Chain names are shown inside the parentheses. **c** Possible mechanisms of chromatin targeting of the DDR complex and Pol V recruitment. The DDR complex is assembled from binding of DRD1 protein to the DR complex. Pol V is then recruited to the DDR complex through interaction with DRD1. However, it is also possible that Pol V is recruited at the same time as DRD1, as a preformed DRD1-Pol V complex. Chromatin targeting could happen during either step and is likely to be mediated, at least, by interaction between DMS3 and methylated DNA binders SUVH2 or SUVH9

First, DRD1 peptide 7 forms three helices (α1, α2, and α3) and binds to DMS3-RDM1 in the DDR′ complex (Fig. 3b, c). Helix α1 is threaded into a symmetric, hydrophobic cleft formed by the RDM1 dimer interface involving the residues Lys121 and Leu128 (Fig. 3e, inset 1). On the other hand, α2 engages in hydrophobic interactions with two CC arms and one hinge from three DMS3 molecules, while α3 interacts with one CC arm and one hinge from two DMS3 molecules (Fig. 3e, inset 2). Notably, DMS3_h_ loop 2, which is freely available in the DR complex now interacts with α3 of DRD1 (Figs. 2e and 3f, blue DMS3_h_(D)). In addition, DMS3_h_ loop 4, which contacts the CC arm in the DR complex now interacts with α2 of DRD1 peptide 7 (Figs. 2e and 3f, blue DMS3_h_(D)). We attempted to model another DRD1 peptide 7 with the same interactions into the complex, however, this resulted in steric clashes between the two α1 helices (Fig. 4a). Therefore, the DDR′ complex most likely contains only one molecule of the peptide.

A second major difference between the DR and DDR′ complexes is that the disordered CC arm of one DMS3_h_ in the DR complex became ordered and visible in the DDR′ complex (i.e., the CC in DMS3_hc2_). This CC arm is the one that engages in the interaction with DRD1 peptide 7 (see above). This suggests that the large conformational change of this CC arm is triggered by DRD1 peptide 7 binding. Intriguingly, the ordered CC arm has a unique conformation that is not observed in the DR complex (Fig. 3d). This DMS3 monomer with newly ordered CC arm was named DMS3_hc2_ (Fig. 3b). Combined with the two DMS3 states already seen in the DR complex, we have three different DMS3 states (DMS3_h_, DMS3_hc1_ and DMS3_hc2_) in the DDR′ complex (Fig. 3d). It is noteworthy that the CC arms from two DMS3_hc1_ are seen in both the DR and DDR′ complexes (Figs. 2e and 3f). These two CC arms do not interact with DRD1 peptide 7. Instead, they are mainly interacting with the hydrophobic pockets of RDM1 (see above) (Fig. 2f).

To confirm the organization within the DDR′ complex, we applied different protein cross-linking reagents—disuccinimidyl suberate (DSS) and adipic dihydrazide (ADH) in combination with 4-(4,6-dimethoxy-1,3,5-triazin-2-yl)-4-methyl-morpholinium chloride (DMTMM)—to the DDR′ complex and analyzed protein–protein interactions using mass spectrometry (MS). We found that the N- and C-coils of DMS3 exhibits extensive internal cross-links as they fold to form the CC arm, RDM1 cross-links mostly to the hinge domain of DMS3, and lastly DRD1 peptide 7 cross-links with the CC of DMS3 (Supplementary Fig. 7 and Supplementary Data 1). These cross-linking-MS (XL-MS) data were in good agreement with our results from the cryoEM analyses.

## Discussion

We report here the structures of the Arabidopsis Pol V recruitment complexes DR (DMS3-RDM1) and DDR′ (DRD1 peptide 7-DMS3-RDM1). The structures of the two complexes are similar at their core—two DMS3 dimers bridged by an RDM1 dimer. We hypothesize that the DR complex might represent an early intermediary complex prior to the formation of the full DDR complex. This is supported by two lines of evidence. First, while expressing the proteins we observed that the DR complex is stable by itself without DRD1. Second, a high enrichment of DMS3 and RDM1 was observed in different mild IP experiments (at 150 mM NaCl) from plant tissues, where DMS3 co-precipitated with higher amounts of RDM1 than DRD1^11^ and RDM1 co-precipitated with higher amounts of DMS3 than DRD1 (Fig. 1a). Combining our structural and biochemical analyses of the DR and DDR′ complex with previous genetic studies paints a possible picture of how the DDR complex may assemble (Fig. 4a).

From the structure, RDM1 dimerization at the core brings together two DMS3 dimers to form a stoichiometry in the DR complex of 4 DMS3:2 RDM1 (Fig. 4a). This is supported by Sasaki et al., who reported that an RDM1 mutant containing L128R and I132R prevented homodimerization and failed to genetically rescue an *rdm1* mutant. However, this mutant maintained its normal ability to interact with DMS3^10^. In the DR complex, two CC arms of DMS3 are interacting with the hydrophobic pockets (HP) of the RDM1 dimer, while the remaining CC arms are free and disordered (Fig. 4a). By comparing structures of the DR with the DDR′ complexes, it appears that as the DDR complex assembles, the binding of DRD1 peptide 7 to the DR complex induces a striking movement of the flexible CC arm of one DMS3_h_ to become ordered and assume the DMS3_hc2_ conformation in the DDR′ complex (Fig. 3b, c, DMS3_hc2_(F)). It is also possible that a larger fragment of DRD1 NTD might stabilize the last missing CC arm of DMS3_h_(D) in the DDR′ complex. The newly ordered CC arm is positioned close to the CC arm of DMS3_hc1_ from the same DMS3 dimer (DMS3_hc1_(E)-DMS3_hc2_(F)) in the DDR′ complex in a state that we refer as a “closed” state (Fig. 4b), and thus the corresponding DMS3 dimer which has a flexible CC arm in the DR complex is referred as an “open” state. A similar large conformational change of the CC arms has also been observed in other SMC complexes (Supplementary Fig. 10, bottom panel) and is thought to regulate their association with DNA^18–20^. However, compared to the SMC complexes, DMS3 exhibits significant differences in the orientation of CC arms (Supplementary Fig. 10). The conformational change observed in DMS3 is also likely to fulfill a different purpose since the inside surface of DMS3 hinge is occupied by RDM1 and contains no significant positively charged patch presumed to bind DNA, as observed in SMC proteins^14,19^. In addition, this conformation change in DMS3 is driven by the binding of DRD1 peptide 7, rather than ATP hydrolysis like in SMC complexes. Given that peptide 7 extensively interacts with the free CC arm, we envisage that the free CC arm in the DR complex is used for DRD1 recruitment to assemble the full DDR complex (Fig. 4a). Further work will be needed to determine whether or not this CC arm by itself is sufficient for this task in the DDR complex.

In both DR and DDR′ complexes, an RDM1 dimer serves as a pillar of the assemblages by providing interacting surfaces for DMS3 dimers. However, it is evident from the DDR′ complex that the RDM1 dimer also participates in DRD1 binding. While helices α2 and α3 of DRD1 peptide 7 engage in interaction mostly with CC arms and a hinge of DMS3, respectively, helix α1 binds to the hydrophobic cleft formed by residues Lys121 and Leu128 at the RDM1 dimer interface (Fig. 3e). Therefore, the RDM1 dimer contributes more than merely preserving integrity of the complexes—it creates a surface for DRD1 interaction. How much this surface alone contributes to DRD1 recruitment remains to be determined. However, our Y2H data suggested that RDM1 by itself could not interact with DRD1 (Supplementary Fig. 1c). Likely, multiple interactions that peptide 7 engages are needed. This is also consistent with our Y3H data showing that DRD1 can only interact when both DMS3 and RDM1 are present (Fig. 1e). Collectively, our data support that the formation of the DR complex is a prerequisite for stable DRD1 binding (Fig. 4a).

We hypothesize that the recruitment of Pol V might be directly bridged by DRD1, given their reported close physical association. Law et al. observed two populations of DRD1 protein in gel filtration experiments with plant nuclear extracts—one co-migrating with the DDR complex and another co-migrating with a large Pol V complex^11^. Supporting this idea, DRD1 was unstable when the Pol V’s catalytic subunit NRPE1 was knocked out in plants^21^. At this point it is unclear if Pol V is recruited directly to the fully assembled DDR complex or if a preformed DRD1-Pol V complex is recruited to the DR complex (Fig. 4c). It is worth noting that the peptide 7 that interacts within the DDR′ complex is a small part of DRD1; >90% of the protein is missing, including most of its N-terminal domain and the C-terminal half containing the SWI2/SNF2 ATPase domain. Thus, in vivo, DRD1 might function beyond bridging Pol V. For instance, the ATPase domain may exert a chromatin remodeling function to ensure either proper Pol V recruitment or to aid in transcriptional processivity^4,22–24^, or it may regulate processing of RNA transcripts^25^. Consistent with this, mutations in the DRD1 ATPase domain abolished RdDM^6,7^.

Gao et al. proposed that the hydrophobic pocket of RDM1 binds to methylated single stranded DNA in a CHH context and suggested that RDM1 thus recruits the RdDM machinery to methylated chromatin^26^. Our cryoEM structures, in contrast, clearly show that both hydrophobic pockets of the RDM1 dimer are occupied with CC domains of DMS3_hc1_ in both DR and DDR′ complexes (Figs. 2e, f, and 3f, right). One explanation for the discrepancy between our observations and the Gao et al. study could be artificial protein binding due to the high protein concentration conditions used in their EMSA experiments (i.e., ~10 μM)^26^. Another possibility is that interactions of the DDR complex with other proteins could promote regulated displacement of the coiled-coil domain to allow DNA binding upon chromatin localization.

Our recent gain-of-function, zinc finger-tethering experiments coupled with loss-of-function RdDM mutants showed that DMS3 and RDM1 are able to trigger RdDM in a *suvh2 suvh9* double mutant, but SUVH9 was unable to trigger RdDM in *dms3* or *rdm1* (or *drd1*) mutants^13^. These experiments clearly place SUVH2/9 upstream of the function of the DDR components. Furthermore, DMS3 and RDM1 were unable to trigger RdDM in either a *drd1* mutant, or in a mutant of Pol V’s catalytic subunit (*nrpe1*), placing the action of DMS3 and RDM1 upstream or at the same step as DRD1 and Pol V. Previous work from Y2H has suggested that DMS3 could be the DDR component that physically interacts with SUVH2/9^4,9,12^. These results, together with the results of the current study, suggest a hypothetical model for the recruitment of Pol V activity in RdDM (Fig. 4c). The model suggests that the DR complex formation is a prerequisite for the recruitment of DRD1 to form the DDR complex. The methylated DNA reader proteins SUVH2/9 could serve as recruiters of the DR and/or DDR complexes to sites previously methylated by RdDM. The DRD1 protein then could serve as a bridge between the DDR and Pol V complexes to ultimately recruit Pol V, which is required to perpetuate additional RdDM.

In summary, in this work we determined the structures of the DR and DDR′ complexes. Conversion from DR to DDR′ complexes could represent a transitional assembly step of the DDR complex leading to Pol V recruitment (Fig. 4c). The structures reveal a shift in conformation of DMS3’s CC domains from an “open” to a “closed” state upon binding of DRD1 to form the DDR complex. Further determination of the recruitment mechanisms of Pol V to the DDR complex and of the DDR to chromatin awaits a structural understanding of the bipartite interactions mediated by DRD1 with both the DR complex and Pol V, as well as those mediated by DMS3 with both the DDR complex and SUVH2/9.

## Methods

### Generation of transgenic RDM1-3xFLAG lines

A cassette containing 3xFLAG was cloned into the pENTR-RDM1 plasmid that contains a genomic fragment of RDM1^13^ to generate pENTR-RDM1-3xFLAG. The RDM1-3xFLAG fragment was transferred to the JP726 destination plasmid^27^ using LR clonase (Invitrogen) to create pEG-RDM1-3xFLAG. This plasmid was introduced into agrobacterium AGL0, which was used to transform *rdm1-3* (FLAG_298G06) plants by the floral dip method.

### Whole-genome bisulfite sequencing

Genomic DNA from Wassilewskija (WS), *rdm1*-3 and two independent lines expressing RDM1-3xFLAG was extracted using the DNAeasy Plant Mini Kit (Qiagen). DNA were sheared to 300 bp with a Covaris S2 (Covaris). Libraries, including bisulfite conversion, were made with the NuGEN Ultralow Methyl-seq kit (NuGEN) and EpiTect Bisulfite kit (Qiagen) following manufacturer’s instructions. WGBS analysis was performed similar as before^13^. In general, raw reads were aligned to TAIR10 genome using BSMAP^28^ with –v 2 (allowing maximal two mismatches) and –n 1 (aligning to both strands). Reads were discarded if there were more than three consecutive methylated CHH sites within the reads (for 50 bp long read)^29^. Methylation levels at each cytosine were calculated as #C/(#C+#T). DMRs between WS and *rdm1* were defined using R package DMRcaller^30^. To define CHH DMRs, parameters were applied as before^31^. Basically, 100 bp bins with more than four cytosines and each cytosines with more than 4 read coverage as well as more than 0.1 difference between *rdm1* and WS were used as cutoff. DMRs within 200 bp of each other were merged for further analysis. Cytosine methylation over *rdm1* hypo CHH DMR were extracted and plotted in R. WGBS tracks displayed in Fig. 1c correspond to wild-type Col-0 samples as described in Stroud et al.^31^.

### Immunoprecipitation and mass spectrometry (IP-MS)

IP-MS was performed as described previously with minor modifications^11^. Two independent RDM1-3xFLAG lines as well as untransformed WS control plants were grown on soil on long-day conditions. Ten grams of inflorescences were collected, grinded in liquid nitrogen and resuspended in 40 mL IP buffer (50 mM Tris pH 7.6, 150 mM NaCl, 5 mM MgCl_2_, 10% glycerol, 0.1% NP40, 0.5 mM DTT, 1 mM PMSF, 1 μg μL^−1^ pepstatin, and 1× Complete EDTA-Free (Sigma)). Tissue was homogenized by douncing, centrifuged for 10 min at 10,000 × *g* at 4 °C and filtered with a 100 μm cell trainer. Two hundred and fifty microliters of Anti-FLAG M2 magnetic beads (Sigma; M8823) were used per sample. Before adding to sample, beads were washed two times in IP buffer, then blocked for 15 min at RT in IP buffer with 5% w/v BSA, and washed two more times with IP Buffer. After incubation at 4 °C for 3 h with rotation, samples were washed five times with IP buffer followed by two washes with IP buffer without NP40. Proteins were eluted three times with 150 μL IP buffer without NP40 but supplemented with 250 μg mL^−1^ 3xFLAG Peptide (Sigma; F4799). The eluted proteins were precipitated with TCA for 30 min on ice and washed with cold acetone and air dried.

TCA precipitated samples were resuspended in 100 mM Tris pH 8.5, 8 M urea and digested by the sequential addition of endoproteinase Lys-C and trypsin as previously described^32^. Digested samples were then fractionated online using reversed-phase chromatography and eluted directed into a Q-Exactive mass spectrometer (ThermoFisher Scientific) where tandem mass spectra were collected using a data-dependent acquisition scheme^33^. Data were subsequently analyzed using the IP2 suite of algorithms which utilizes ProLuCID for database searching and DTASelect for filtering using decoy-database estimated false discovery rates^34,35^. Comparison of peptide and protein identifications across samples were performed using in-house scripts.

### Chromatin immunoprecipitation (ChIP)

ChIP was performed as described previously with minor modifications^13^. Briefly, 3 g of inflorescences from RDM1-3xFLAG plants described in this work, as well as DMS3-3xFLAG and DRD1-3xFLAG plants described in Law et al.^11^ grown on soil under long day conditions, were grinded in liquid nitrogen and fixed in Nuclei Isolation Buffer containing 1% v/v formaldehyde. Fixation reaction was stopped with addition of glycine, nuclei were extracted, and chromatin sonicated using a Bioruptor Plus (Diagenode). Chromatin was immunoprecipitated with 5 μg of anti-FLAG M2 antibody (Sigma; F1804) overnight at 4 °C with rotation and captured with a 1:1 mix of protein G and protein A Dynabeads (Invitrogen; 10002D and 10004D) for 3 h at 4 °C with rotation. Complexes were washed once with Low Salt Buffer, once with High Salt Buffer, once with LiCl buffer and twice with EB buffer, then eluted in elution buffer twice at 65 °C for 20 min. Cross-link reversal was done overnight at 65 °C, followed by proteinase K treatment 4 h at 45 °C. DNA was purified using phenol:chloroform and precipitated overnight at −20 °C in NaOAc, ethanol and Glycoblue (Invitrogen). Sequencing libraries were prepared using the Ovation Ultra Low System V2 1-16 kit (NuGEN), following the manufacturer’s instructions.

For data analysis, raw reads were aligned to the Arabidopsis reference genome (TAIR10) with Bowtie (v1.0.0)^36^, allowing only uniquely mapping reads with at most two mismatches, and duplicated reads were removed with Samtools 0.1.19^37^. Pol V peaks were called comparing Pol V antibody ChIP against no antibody control ChIP described in Liu et al.^38^, using MACS2 with *q*-value 0.05 (v 2.1.1)^39^. For ChIP-seq metaplot and heatmap, the summit of peaks with more than twofolds of enrichment were used and plots were generated using NGSplot (v 2.41.4)^40^. BamCompare (deepTools 2.0)^41^ was used to generate the ChIP-seq tracks that show the log_2_ ratio of FLAG ChIP-seq signal in DRD1, DMS3, and RDM1 transgenic lines over FLAG ChIP-seq signal in untransformed Col0 control, or the log2 ratio of anti-Pol V ChIP-seq signal to No antibody control. IGV (2.4.16) was used to visualize the resulting files.

### Yeast two- and three-hybrid

For the Yeast experiments, the CDS sequences of RDM1, DMS3, and DRD1, as well as the DRD1 deletions reported were cloned in the pENTR/D plasmid (Invitrogen) and sub-cloned into pDEST22 and pDEST32 destination plasmids (Invitrogen) using LR clonase (Invitrogen). For Y3H experiments, RDM1 CDS was cloned in a modified pYES-DEST52 (Invitrogen), where the GAL1 promoter was replaced by the constitutive ADH1 promoter from pDEST22/32.

Yeast transformation was done following the ProQuest Two-Hybrid System (Invitrogen) according to manufacturer’s instructions and using the MaV203 yeast strain. Four independent colonies per condition were grown in medium with different concentration of 3-amino-1,2,4-triazole (3-AT, Sigma).

### Protein expression and purification

Arabidopsis RDM1 and DMS3 were cloned into pCDFDuet-1 (EMD Millipore), with 6xHis tag on the N-terminus of RDM1. Arabidopsis DRD1 Peptide 7 was cloned into pET21b (EMD Millipore), with Strep II tag on the C-terminus of DRD1. Plasmids were transformed into Rosetta2 (DE3) *E. coli* (EMD Millipore) for protein expression—pCDFDuet-1 for the DR complex; pCDFDuet-1 and pET21b for the DDR′ complex. A single colony was inoculated in a 500–1000 mL LB culture shaken at 37 °C. Once O.D._600_ reached 0.4–0.6, the culture was cooled down to 16 °C and IPTG was added to the final concentration of 0.1 mM. The culture was continued to grow at 16 °C in a shaker overnight (16–20 h). Cells were harvested at 5000 rpm for 15 min at 4 °C and washed once with PBS. Cell pellet was either used directly for protein purification or stored at −80 °C for a later use.

The pellet was resuspended in 30–40 mL of buffer A containing 20 mM HEPES-KOH pH 7.5, 150 mM NaCl, 10% v/v glycerol, 3 mM DTT. For His tag affinity pulldown, 50 mM imidazole was also added to buffer A to reduce non-specific binding to the resin. The suspension was supplemented with 1× Complete protease inhibitor cocktail (Roch), 1 mM PMSF, 1 mM benzamidine, and 1.5 mg mL^−1^ egg white lysozyme, and incubated on ice for 30 min. Afterward, the suspension was sonicated at 4 °C to break cells. The suspension was spun down at 11,000 × *g* for 1 h at 4 °C to remove debris. The supernatant was recovered and filtered through a 0.22 μm PES membrane (EMD Millipore). The DR complex was purified using a 5-mL His Trap HP (GE), while the DDR′ complex was purified using a 5-mL Strep Trap HP (GE), following manufacturers’ recommendation. Equilibration and wash buffer for His Trap HP contained 20 mM HEPES-KOH pH 7.5, 150 mM NaCl, 10% v/v glycerol, 2 mM DTT, and 50 mM imidazole; elution buffer was supplemented with 500 mM imidazole. Equilibration and wash buffer for Strep Trap HP contained 20 mM HEPES-KOH pH 7.5, 150 mM NaCl, 10% v/v glycerol, and 3 mM DTT; elution buffer contained 50 mM Tris-HCl pH 7.5, 150 mM NaCl, 1 mM EDTA, and 2.5 mM desthiobiotin. Eluted complexes were concentrated at 4 °C using Amicon Ultra-15 with 100 kDa cutoff (EMD Millipore). A HiLoad 16/600 Superdex 200 pg column (GE) was equilibrated with buffer containing 20 mM HEPES-KOH pH 7.5, 150 mM NaCl on NGC Quest 10 Plus FPLC system (Bio-Rad). The concentrated samples were loaded and run on the column at a flow rate of 0.8–1.0 ml min^−1^. Fractions were collected and analyzed on SDS-PAGE stained with Imperial protein stain (ThermoFisher Scientific). Peak fractions were combined and concentrated using Amicon Ultra-15 with 100 kDa cutoff. Concentrated complex was either used directly for EM or stored at −80 °C for later uses (20% v/v glycerol was added before storage).

### CryoEM sample preparation and imaging

For cryoEM sample optimization, an aliquot of 2.5 μL of sample at a concentration of 0.45–0.5 mg mL^−1^ was applied onto a glow-discharged holey carbon-coated copper grid (300 mesh, QUANTIFOIL® R 1.2/1.3). The grid was blotted with Grade 595 filter paper (Ted Pella) and flash-frozen in liquid ethane with an FEI Mark IV Vitrobot. An FEI TF20 cryoEM instrument was used to screen grids. CryoEM grids with optimal particle distribution and ice thickness were obtained by varying the gas source (air using PELCO easiGlow^TM^, target vacuum of 0.37 mbar, target current of 15 mA; or H_2_/O_2_ using Gatan Model 950 advanced plasma system, target vacuum of 70 mTorr, target power of 50 W) and time for glow discharge, the volume of applied samples, chamber temperature and humidity, blotting time and force, as well as drain time after blotting. Our best grids for DR complex were obtained with 20 s glow discharge using H_2_/O_2_ and with the Vitrobot sample chamber set at 10 °C temperature, 100% humidity, 10 s blotting time, −8 blotting force, and 2 s drain time. The best grids for DDR′ complex were obtained with 20 s glow discharge using H_2_/O_2_ and with the Vitrobot sample chamber set at 10 °C temperature, 100% humidity, 11 s blotting time, −5 blotting force, and 1 s drain time.

Optimized cryoEM grids were loaded into an FEI Titan Krios electron microscope with a Gatan Imaging Filter (GIF) Quantum LS device and a post-GIF K2 Summit direct electron detector. The microscope was operated at 300 kV with the GIF energy-filtering slit width set at 20 eV. Movies were acquired with Leginon^42^ by electron counting in super-resolution mode at a pixel size of 0.535 Å/pixel (DR complex) or counting mode at a pixel size of 1.07 Å/pixel (DDR′ complex). A total number of 40 frames were acquired in 8 s for each movie, giving a total dose of ~50 e^−^/Å^2^/movie.

### Drift correction for movie frames

Frames in each movie were aligned for drift correction with the GPU-accelerated program MotionCor2^43^. The first frame was skipped during drift correction due to concern of more severe drift/charging of this frame. Two averaged micrographs, one with dose weighting and the other without dose weighting, were generated for each movie after drift correction. The averaged micrographs have a calibrated pixel size of 1.07 Å on the specimen scale. The averaged micrographs without dose weighting were used only for defocus determination and the averaged micrographs with dose weighting were used for all other steps of image processing.

### Structure determination for the DR complex

For the DR complex, the defocus values of the averaged micrographs were determined by CTFFIND4^44^ to be ranging from −1.5 to −2.5 μm. Initially, a total of 1,543,489 particles were automatically picked from 2513 averaged micrographs without reference using Gautomatch (http://www.mrc-lmb.cam.ac.uk/kzhang). The particles were boxed out in dimensions of 256 × 256 square pixels and binned to 128 × 128 square pixels (pixel size of 2.14 Å) before further processing by the cryoSPARC v1^45^. Several iterations of reference-free 2D classification were subsequently performed to remove “bad” particles (i.e., classes with fuzzy or un-interpretable features), yielding 1,148,692 good particles. The resulting particles were subjected to ab initio reconstruction and 3D heterogeneous classification with three classes in cryoSPARC v1. Only one class exhibiting good model features was kept, which contained 31.9% of all particles (Supplementary Fig. 3a). Particles with class probability lower than 0.9 were removed from this good class. We re-centered the remaining particles of this good class and removed duplications based on the unique index of each particle. The resulting particles were un-binned to 256 × 256 square pixels (pixel size of 1.07 Å) for the final refinement.

The 314,414 un-binned, unique particles (20.4% of all particles) resulting from the heterogeneous classification were subjected to a final step of 3D auto-refinement in RELION2.1 (Supplementary Fig. 3a). The two half maps from this auto-refinement step were subjected to RELION’s standard post-processing procedure. The final map of the DR complex has an average resolution of 3.6 Å based on RELION’s gold-standard FSC (see below).

### Structure determination for the DDR′ complex

For the DDR′ complex, the defocus values of the averaged micrographs were determined by CTFFIND4 to be ranging from −1.5 to −2.5 μm. Initially, a total of 2,482,628 particles were automatically picked from 4383 averaged micrographs without reference using Gautomatch. The particles were boxed out in dimensions of 240 × 240 square pixels (pixel size of 1.07 Å) for processing by the cryoSPARC v1. Several iterations of reference-free 2D classification were subsequently performed to remove “bad” particles (i.e., classes with fuzzy or un-interpretable features), yielding 1,767,986 good particles. The resulting particles were subjected to ab initio reconstruction and 3D heterogeneous classification with five classes in cryoSPARC v1. Only one class exhibiting good model features was kept, which contained 25.5% of all particles (Supplementary Fig. 3a). We re-centered the particles of this good class and removed duplications based on the unique index of each particle for the final refinement (Supplementary Fig. 4a).

The 620,248 un-binned, unique particles (25.0% of all particles) resulting from the heterogeneous classification were subjected to a final step of 3D auto-refinement in RELION2.1 (Supplementary Fig. 4a). The two half maps from this auto-refinement step were subjected to RELION’s standard post-processing procedure. The final map of the DDR′ complex has an average resolution of 3.5 Å based on RELION’s gold-standard FSC (see below).

### Resolution assessment

All resolutions reported above are based on the “gold-standard” FSC 0.143 criterion^46^. FSC curves were calculated using soft spherical masks and high-resolution noise substitution was used to correct for convolution effects of the masks on the FSC curves^47^. Prior to visualization, all maps were sharpened by applying a negative *B* factor which was estimated using automated procedures^48^. Local resolution was estimated using ResMap^49^. The overall quality of the maps for DR and DDR′ complexes is presented in Supplementary Fig. 3b-d and Supplementary Fig. 4b–d, respectively. Data collection and reconstruction statistics are summarized in Table 1.

### Model building and refinement

To aid subunit assignment and model building, we took advantage of the reported RDM1 structure (PDB code: 3GAN), which was fitted into the central region of DR complex density map by UCSF Chimera^50^. We manually adjusted its side chain conformation and, when necessary, moved the main chains to match the density map using COOT^51^. Next, we built the atomic model for DMS3 de novo. Sequence assignment was mainly guided by visible densities of amino acid residues with bulky side chains, such as Trp, Tyr, Phe, and Arg. Other residues including Gly and Pro also helped the assignment process. Unique patterns of sequence segments containing such residues were utilized for validation of residue assignment.

For the DDR′ complex, the DR complex model was rigidly fitted into the density map of DDR′ and manually adjusted using COOT. This enabled us to identify extra densities for DRD1 peptide 7 and one coiled-coil arm of DMS3 absent in the DR complex, of which the atomic models were build de novo with similar methods as mentioned above.

The atomic models were refined using PHENIX in real space^52^ with secondary structure and geometry restraints. Refinement statistics of DR and DDR′ were summarized in Table 1. These two models were also evaluated based on Molprobity scores^53^ and Ramachandran plots (Table 1). Model/map FSC validation was shown in Supplementary Figs. 3e and 4e. Representative densities for the proteins and RNA are shown in Supplementary Fig. 5. All structure-related images in this paper were generated using UCSF Chimera and ChimeraX^54^.

### His tag affinity pulldown

Rosetta2 (DE3) *E. coli* expressing pCDFDuet-1-His-RDM1/DMS3 or pCDFDuet-1-His-RDM1/DMS3 G339E were induced for protein expression and cell lysate preparation as above. Fifty microliters of magnetic Ni-NTA agarose suspension (QIAgen) was equilibrated with wash buffer (20 mM HEPES pH 7.5, 200 mM NaCl, 10% v/v glycerol, 50 mM imidazole, 0.05% v/v Tween20, 2 mM DTT, and 1 mM PMSF. Seven hundred microliters of the cell lysate was incubated with the beads at 4 °C with rotation. The supernatant was removed, and the beads were washed four times with 1000 μL of wash buffer at 4 °C with rotation. Bound proteins were eluted twice with 20 μL of elution buffer (20 mM HEPES pH 7.5, 150 mM NaCl, 10% v/v glycerol, 500 mM imidazole, and 2 mM DTT) at 4 °C for 5 min. Eluates were combined. Samples were analyzed on SDS-PAGE, stained with Imperial protein stain, and scanned and quantified on Odyssey CLx imager (LI-COR Biosciences) using 700 and 800-nm channels. Protein bands in the eluates were normalized to those in the inputs, then values of DMS3/RDM1 were calculated. Averages from two experiments are shown in Fig. 2c.

For gel filtration analysis of DMS3 WT and G339E (Fig. 2c), Rosetta2 (DE3) *E. coli* expressing either pCDF-His-DMS3 or pCDF-His-DMS3 G339E were induced for protein expression and cell lysate preparation as above. Cell lysates corresponding to 100-mL culture were passed through 300 μL of packed Ni-NTA agarose (QIAgen) at 4 °C. The beads were washed extensively with wash buffer (20 mM HEPES-KOH pH 7.5, 150 mM NaCl, 10% v/v glycerol, 2 mM DTT and 50 mM imidazole) and eluted twice with 100 μL of elution buffer (wash buffer supplemented with 500 mM imidazole) at 4 °C for 10 min. The eluates were combined and analyzed on an analytical Superdex 200 10/300 GL (GE) at flow rate of 0.5 mL min^−1^, which was equilibrated with gel filtration buffer (20 mM HEPES-KOH pH 7.5, 150 mM NaCl).

### Protein cross-linking

Isotopic DSS (DSS-H12/D12) and ADH (ADH-H8/D8) were used to cross-link the DDR′ complex. DSS cross-links between primary amines on lysine residues and the proteins’ N termini, while ADH cross-links among acidic amino residues (Asp and Glu) but relies on the coupling reagent DMTMM to activate carboxylic groups during the cross-linking reaction. DMTMM can also directly cross-link an acidic residue to lysine by itself. Therefore, in an ADH/DMTMM reaction, two types of chemistry will be generated—ADH-mediated and DMTMM-mediated cross-linking (the latter is called zero link; ZL)—which can be distinguished by MS analysis. A typical molar ratio of ADH to DMTMM is 1:1 in a cross-linking reaction^55^. Optimal concentration of the cross-linkers and cross-linking conditions (time and temperature) were empirically determined using SDS-PAGE and protein staining. In the cross-linking experiments, 50 μg of DDR′ complex was cross-linked either with 15 mM of ADH (Creative Molecules Inc.) and DMTMM (Sigma) or 0.5 mM of DSS (Creative Molecules Inc.) at room temperature for 10 and 30 min, respectively. The reactions were then quenched with 100 mM sodium acetate/Tris, pH 7.0 or 50 mM ammonium bicarbonate, respectively, at 37 °C for 30 min. Samples were stored at −80 °C until MS analysis.

### Sample processing for MS analysis

Following the cross-linking and quenching steps, samples were processed essentially as described previously^56^. Disulfide bonds were reduced by addition of tris(2-carboxyethyl)phosphine, free thiol groups were alkylated with iodoacetamide and proteins were digested by endoproteinase Lys-C (Wako, 1:100, 3 h at 37 °C) and trypsin (Promega, 1:50, overnight at 37 °C). Digests were purified by solid-phase extraction (Waters Sep-Pak tC18) and fractionated by size-exclusion chromatography (SEC) using a Superdex Peptide PC 3.2/300 column (GE Healthcare)^56^.

### Liquid chromatography-tandem mass spectrometry (LC-MS/MS)

Duplicate injections of three SEC fractions were analyzed with an Easy-nLC 1200 system coupled to an Orbitrap Fusion Lumos mass spectrometer (both ThermoFisher Scientific). Chromatographic separation was achieved using an Acclaim PepMap RSLC C18 column (25 cm × 75 μm, 2 μm particle size) (ThermoFisher Scientific) and a gradient of 11–40% B in 60 min, where mobile phases A = water/acetonitrile/formic acid, 95:5:0.15, v/v/v and B = acetonitrile/water/formic acid, 80:20:0.15, v/v/v. The flow rate was set to 300 nL min^−1^. The mass spectrometer was operated in top speed mode with a cycle time of 3 s, consisting of an MS scan in the Orbitrap analyzer at 120,000 resolution, followed by MS/MS scans in the linear ion trap in rapid scan mode. Fragmentation was performed using collision induced dissociation in the linear ion trap with an isolation width of 1.2 *m*/*z* and a normalized collision energy of 35%. Only precursors with charge states between 3+ and 7+ were selected for fragmentation, and monoisotopic peak selection was set to “peptide” mode. Dynamic exclusion (±10 ppm, 30 s, 1 repeat count) was enabled.

### Identification of cross-linked peptides with xQuest

xQuest^56,57^ was used to identify cross-linked peptides from MS/MS data, using a database containing the sequences of the three target proteins plus seven contaminant proteins identified from a regular protein identification search using Mascot (MatrixScience): four human keratins and three *E. coli* proteins. The main search settings for xQuest were Enzyme = trypsin, maximum number of missed cleavages = 2, mass tolerance = 15 ppm for MS data, and 0.2/0.3 Da for MS/MS data. Search results were further filtered according to the following settings: mass error = −6 to +1 ppm, %TIC sub-score ≥ 0.1 for DSS and ADH data, and ≥0.15 for ZL data, delta score ≥ 0.9. In addition, the following xQuest score cut-offs were applied: ≥18 for DSS and ADH data and ≥23 for ZL data. Candidate cross-links fulfilling these criteria were manually evaluated and only identifications with at least four bond cleavages overall or three consecutive bond cleavages per peptide were retained for the final list of identifications. No further attempts for site localization were made. All cross-linked peptides are listed in Supplementary Data 1.

### Reporting summary

Further information on research design is available in the Nature Research Reporting Summary linked to this article.

## Supplementary information

Supplementary Information

Description of Additional Supplementary Files

Supplementary Data 1

Reporting Summary

## Data Availability

The whole-genome bisulfite sequencing and the ChIP sequencing data that support the findings of this study are available in Gene Expression Omnibus (GEO) with accession number GSE130319 and GSE130290, respectively. The atomic models and cryoEM density maps in this study are available from both PDB and EMDB with accession number 6OIS (DR), 6OIT (DDR′) and EMDB-20080 (DR), EMDB-20081 (DDR′), respectively. The proteomics data of the IP-MS experiment (Fig. 1a) are available from the MassIVE data repository (https://massive.ucsd.edu) with the dataset identifier MSV000084088. The cross-linking-MS data that support the findings in this study are available from the ProteomeXchange Consortium (proteomecentral.proteomexchange.org) via the PRIDE partner repository^58^ with the dataset identifier PXD013470. All other relevant data supporting the key findings of this study are available within the article and its Supplementary Information files or from the corresponding authors upon reasonable request. A reporting summary for this Article is available as a Supplementary Information file.

## References

[1] Law JA, Jacobsen SE (2010). Establishing, maintaining and modifying DNA methylation patterns in plants and animals. Nat. Rev. Genet..

[2] Matzke MA, Mosher RA (2014). RNA-directed DNA methylation: an epigenetic pathway of increasing complexity. Nat. Rev. Genet..

[3] Zhou M, Law JA (2015). RNA Pol IV and V in gene silencing: Rebel polymerases evolving away from Pol II’s rules. Curr. Opin. Plant Biol..

[4] Matzke MA, Kanno T, Matzke AJM (2015). RNA-directed DNA methylation: the evolution of a complex epigenetic pathway in flowering plants. Annu Rev. Plant Biol..

[5] Zhong X (2012). DDR complex facilitates global association of RNA polymerase V to promoters and evolutionarily young transposons. Nat. Struct. Mol. Biol..

[6] Kanno T (2004). Involvement of putative SNF2 chromatin remodeling protein DRD1 in RNA-directed DNA methylation. Curr. Biol..

[7] Kanno T (2008). A structural-maintenance-of-chromosomes hinge domain-containing protein is required for RNA-directed DNA methylation. Nat. Genet..

[8] Allard STM (2005). Structure at 1.6 Å resolution of the protein from gene locus At3g22680 from *Arabidopsis thaliana*. Acta Crystallogr Sect. F. Struct. Biol. Cryst. Commun..

[9] Johnson LM (2014). SRA- and SET-domain-containing proteins link RNA polymerase V occupancy to DNA methylation. Nature.

[10] Sasaki T, Lorković ZJ, Liang S-C, Matzke AJM, Matzke M (2014). The ability to form homodimers is essential for RDM1 to function in RNA-directed DNA methylation. PLoS ONE.

[11] Law JA (2010). A protein complex required for polymerase V transcripts and RNA-directed DNA methylation in *Arabidopsis*. Curr. Biol..

[12] Liu Z-W (2014). The SET domain proteins SUVH2 and SUVH9 are required for Pol V occupancy at RNA-directed DNA methylation loci. PLoS Genet..

[13] Gallego-Bartolomé J (2019). Co-targeting RNA polymerases IV and V promotes efficient de novo DNA methylation in *Arabidopsis*. Cell.

[14] Hirano T (2006). At the heart of the chromosome: SMC proteins in action. Nat. Rev. Mol. Cell Biol..

[15] Haering CH, Löwe J, Hochwagen A, Nasmyth K (2002). Molecular architecture of SMC proteins and the yeast cohesin complex. Mol. Cell.

[16] Li Y, Schoeffler AJ, Berger JM, Oakley MG (2010). The crystal structure of the hinge domain of the *Escherichia coli* structural maintenance of chromosomes protein MukB. J. Mol. Biol..

[17] Ku B, Lim J-H, Shin H-C, Shin S-Y, Oh B-H (2010). Crystal structure of the MukB hinge domain with coiled-coil stretches and its functional implications. Proteins.

[18] Uhlmann F (2016). SMC complexes: from DNA to chromosomes. Nat. Rev. Mol. Cell Biol..

[19] Soh Y-M (2015). Molecular basis for SMC rod formation and its dissolution upon DNA binding. Mol. Cell.

[20] Diebold-Durand M-L (2017). Structure of full-length SMC and rearrangements required for chromosome organization. Mol. Cell.

[21] Lahmy S (2016). Evidence for ARGONAUTE4-DNA interactions in RNA-directed DNA methylation in plants. Genes Dev..

[22] Dürr H, Flaus A, Owen-Hughes T, Hopfner K-P (2006). Snf2 family ATPases and DExx box helicases: differences and unifying concepts from high-resolution crystal structures. Nucleic Acids Res..

[23] Sprouse RO, Brenowitz M, Auble DT (2006). Snf2/Swi2-related ATPase Mot1 drives displacement of TATA-binding protein by gripping DNA. EMBO J..

[24] Ryan DP, Owen-Hughes T (2011). Snf2-family proteins: chromatin remodellers for any occasion. Curr. Opin. Chem. Biol..

[25] Wang Z (2018). SWI2/SNF2 ATPase CHR2 remodels pri-miRNAs via Serrate to impede miRNA production. Nature.

[26] Gao Z (2010). An RNA polymerase II- and AGO4-associated protein acts in RNA-directed DNA methylation. Nature.

[27] Johnson LM, Law JA, Khattar A, Henderson IR, Jacobsen SE (2008). SRA-domain proteins required for DRM2-mediated de novo DNA methylation. PLoS Genet..

[28] Xi Y, Li W (2009). BSMAP: whole genome bisulfite sequence MAPping program. BMC Bioinforma..

[29] Cokus SJ (2008). Shotgun bisulphite sequencing of the *Arabidopsis* genome reveals DNA methylation patterning. Nature.

[30] Catoni M, Tsang JM, Greco AP, Zabet NR (2018). DMRcaller: a versatile R/Bioconductor package for detection and visualization of differentially methylated regions in CpG and non-CpG contexts. Nucleic Acids Res..

[31] Stroud H, Greenberg MVC, Feng S, Bernatavichute YV, Jacobsen SE (2013). Comprehensive analysis of silencing mutants reveals complex regulation of the *Arabidopsis* methylome. Cell.

[32] Heinzelman P, Powers DN, Wohlschlegel JA, John V (2019). Shotgun proteomic profiling of bloodborne nanoscale extracellular vesicles. Methods Mol. Biol..

[33] Kelstrup CD, Young C, Lavallee R, Nielsen ML, Olsen JV (2012). Optimized fast and sensitive acquisition methods for shotgun proteomics on a quadrupole orbitrap mass spectrometer. J. Proteome Res..

[34] Xu T (2015). ProLuCID: an improved SEQUEST-like algorithm with enhanced sensitivity and specificity. J. Proteom..

[35] Tabb DL, McDonald WH, Yates JR (2002). DTASelect and Contrast: tools for assembling and comparing protein identifications from shotgun proteomics. J. Proteome Res..

[36] Langmead B, Trapnell C, Pop M, Salzberg SL (2009). Ultrafast and memory-efficient alignment of short DNA sequences to the human genome. Genome Biol..

[37] Li H (2009). The sequence alignment/map format and SAMtools. Bioinformatics.

[38] Liu W (2018). RNA-directed DNA methylation involves co-transcriptional small-RNA-guided slicing of polymerase V transcripts in *Arabidopsis*. Nat. Plants.

[39] Zhang Y (2008). Model-based analysis of ChIP-Seq (MACS). Genome Biol..

[40] Shen L, Shao N, Liu X, Nestler E (2014). ngs.plot: Quick mining and visualization of next-generation sequencing data by integrating genomic databases. BMC Genom..

[41] Ramírez F, Dündar F, Diehl S, Grüning BA, Manke T (2014). deepTools: a flexible platform for exploring deep-sequencing data. Nucleic Acids Res..

[42] Carragher B (2000). Leginon: an automated system for acquisition of images from vitreous ice specimens. J. Struct. Biol..

[43] Zheng SQ (2017). MotionCor2: anisotropic correction of beam-induced motion for improved cryo-electron microscopy. Nat. Methods.

[44] Rohou A, Grigorieff N (2015). CTFFIND4: Fast and accurate defocus estimation from electron micrographs. J. Struct. Biol..

[45] Punjani A, Rubinstein JL, Fleet DJ, Brubaker M (2017). A. cryoSPARC: algorithms for rapid unsupervised cryo-EM structure determination. Nat. Methods.

[46] Scheres SHW, Chen S (2012). Prevention of overfitting in cryo-EM structure determination. Nat. Methods.

[47] Chen S (2013). High-resolution noise substitution to measure overfitting and validate resolution in 3D structure determination by single particle electron cryomicroscopy. Ultramicroscopy.

[48] Rosenthal PB, Henderson R (2003). Optimal determination of particle orientation, absolute hand, and contrast loss in single-particle electron cryomicroscopy. J. Mol. Biol..

[49] Kucukelbir A, Sigworth FJ, Tagare HD (2014). Quantifying the local resolution of cryo-EM density maps. Nat. Methods.

[50] Pettersen EF (2004). UCSF Chimera-a visualization system for exploratory research and analysis. J. Comput Chem..

[51] Emsley P, Lohkamp B, Scott WG, Cowtan K (2010). Features and development of Coot. Acta Crystallogr. D. Biol. Crystallogr..

[52] Adams PD (2010). PHENIX: a comprehensive Python-based system for macromolecular structure solution. Acta Crystallogr. D Biol. Crystallogr..

[53] Chen VB (2010). MolProbity: all-atom structure validation for macromolecular crystallography. Acta Crystallogr. D. Biol. Crystallogr..

[54] Goddard TD (2018). UCSF ChimeraX: meeting modern challenges in visualization and analysis. Protein Sci..

[55] Leitner A (2014). Chemical cross-linking/mass spectrometry targeting acidic residues in proteins and protein complexes. Proc. Natl Acad. Sci. USA.

[56] Leitner A, Walzthoeni T, Aebersold R (2014). Lysine-specific chemical cross-linking of protein complexes and identification of cross-linking sites using LC-MS/MS and the xQuest/xProphet software pipeline. Nat. Protoc..

[57] Rinner O (2008). Identification of cross-linked peptides from large sequence databases. Nat. Methods.

[58] Perez-Riverol Y (2019). The PRIDE database and related tools and resources in 2019: improving support for quantification data. Nucleic Acids Res..

[59] Florens L (2006). Analyzing chromatin remodeling complexes using shotgun proteomics and normalized spectral abundance factors. Methods.

[60] Notredame C, Higgins DG, Heringa J (2000). T-Coffee: a novel method for fast and accurate multiple sequence alignment. J. Mol. Biol..

