# Supplementary material for: CryoEM structures of Arabidopsis DDR complexes involved in RNA-directed DNA methylation
Source: Nat Commun. 2019 Sep 2;10:3916. doi: 10.1038/s41467-019-11759-9 (PMC6718625; doi:10.1038/s41467-019-11759-9)
Supplement: Supplementary file 1 — Supplementary Information [file 41467_2019_11759_MOESM1_ESM.pdf]

SUPPLEMENTARY INFORMATION

CryoEM Structures of Arabidopsis DDR Complexes Involved in RNA-directed DNA Methylation

Wongpalee *et al.*

Supplementary Figure 1

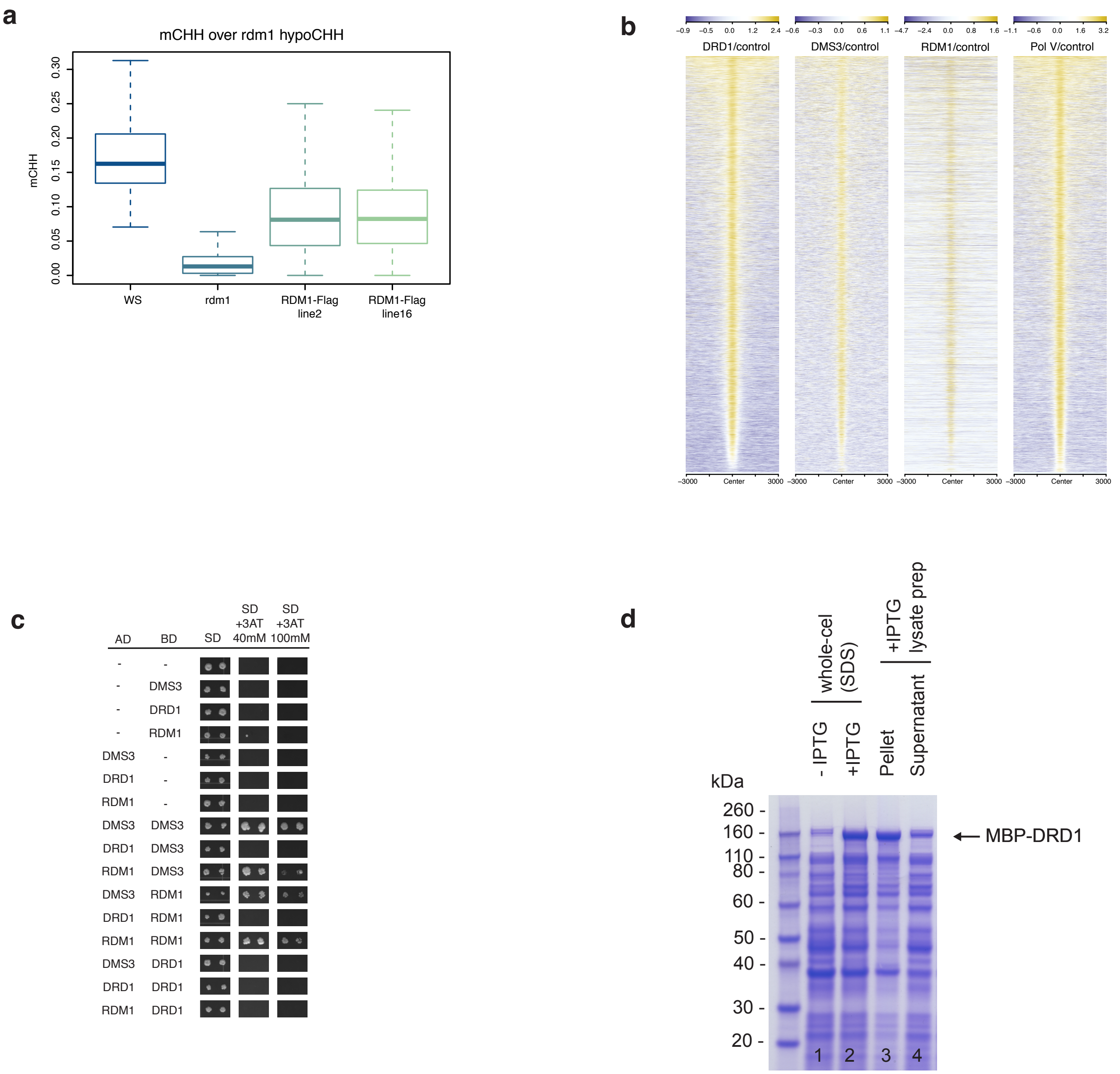

Supplementary Fig. 1. *In vivo* and *in vitro* interaction among DDR proteins.

a, Complementation analysis showing rescue of *rdm1* mutant by RDM1-Flag transgene. From WGBS, regions hypomethylated in *rdm1*-3 mutant (as compared to wild-type WS control) are analyzed for CHH methylation levels. Data from WS, *rdm1*-3, and two independent RDM1-Flag-rescued *rdm1*-3 lines are shown. For boxplots, the boxes span from the 25th (lower margin) to 75th (upper margin) percentiles, while middle lines represent the median; the error bars represent the minimum and maximum points within 1.5x interquartile range. **b**, Heatmap showing DRD1, DMS3, RDM1 and Pol V ChIP-seq signals over Pol V peaks. Signals correspond to the ratio of Flag ChIP-seq enrichment in transgenic lines over untransformed control for DDR, or the ratio of anti-Pol V ChIP-seq enrichment over No antibody control for Pol V. Color scales are shown as $\log_2$ fold change of ChIP over control. **c**, Y2H experiment showing binary interaction between the RDM1, DMS3 and DRD1. BD: GAL4 binding domain, AD: GAL4 activation domain. Growth of two independent colonies in minimal medium supplemented with increasing concentrations of 3-AT is shown. **d**, Protein-stained SDS-PAGE showing protein profile in each condition.
E. coli are transeformed with a plasmid expressing MBP-DRD1. Cells are induced without or with IPTG overnight at 16 ºC (lane 1 and 2, respectively). To test solubility of MBP-DRD1, lysate is prepared from the +IPTG culture, then subjected to centrifugation at 16,000 g at 4 ºC for 30 min. Pellet and supernatant are then analyzed in SDS-PAGE (lane 3 and 4). The uncropped image is shown in Supplementary Fig. 11b.

Supplementary Figure 2

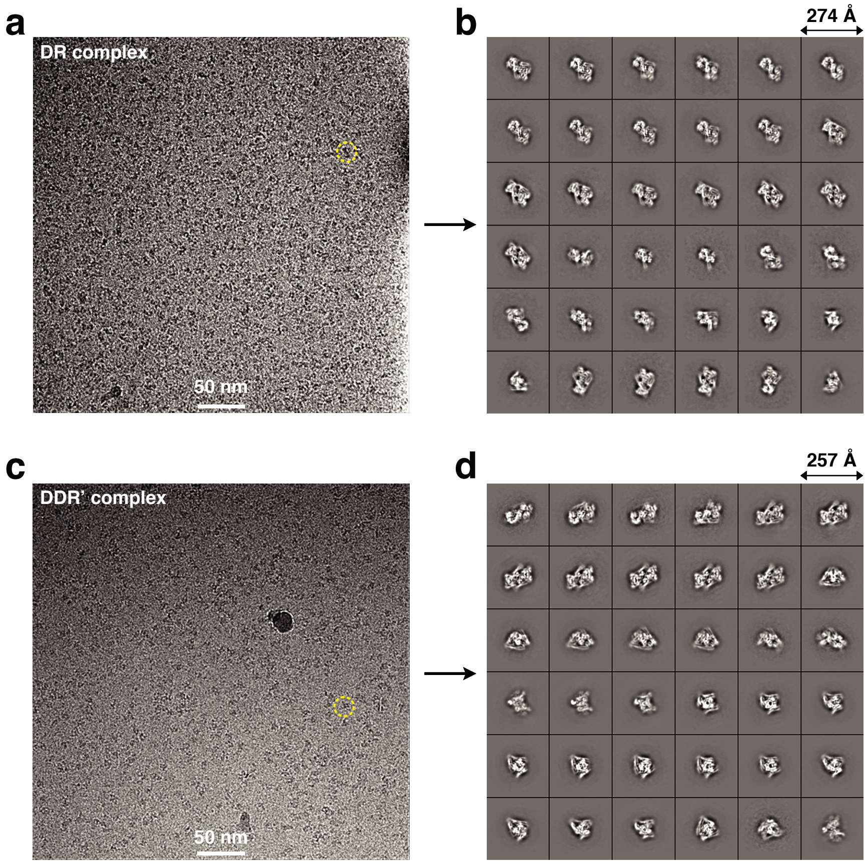

Supplementary Fig. 2. CryoEM images and 2D classification of the DR and DDR' complex.

a, A drift-corrected cryoEM micrograph of the DR complex. A representative particle is shown in a yellow dotted circle. **b**, Representative 2D class averages of the DR complex obtained in RELION. **c**, A drift-corrected cryoEM micrograph of the DDR' complex. A representative particle is shown in a yellow dotted circle. **d**, Representative 2D class averages of the DDR' complex obtained in RELION.

Supplementary Figure 3

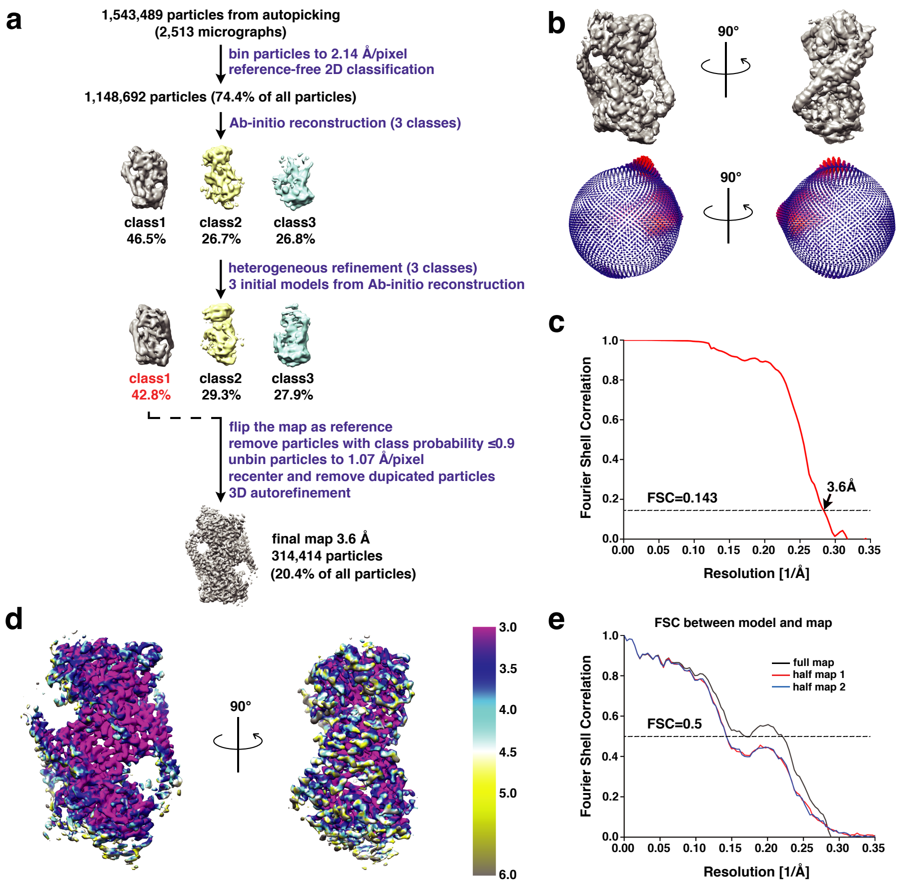

Supplementary Fig. 3. CryoEM structural determination and evaluation for the DR complex.

a, Data processing workflow. Please refer to Materials and Methods for more details. **b**, Angular distribution for all particles used for the final 3.6 Å map of the DR complex. **c**, Fourier shell correlation (FSC) as a function of spatial frequency demonstrating the resolution for the final reconstruction of the DR complex. **d**, Resmap local resolution estimation. **e**, FSC coefficients as a functional of spatial frequency between model and cryoEM density maps. The generally similar appearances between the FSC curves obtained with half maps with (red) and without (blue) model refinement indicate that the refinement of the atomic coordinates did not suffer from severe over-fitting.

Supplementary Figure 4

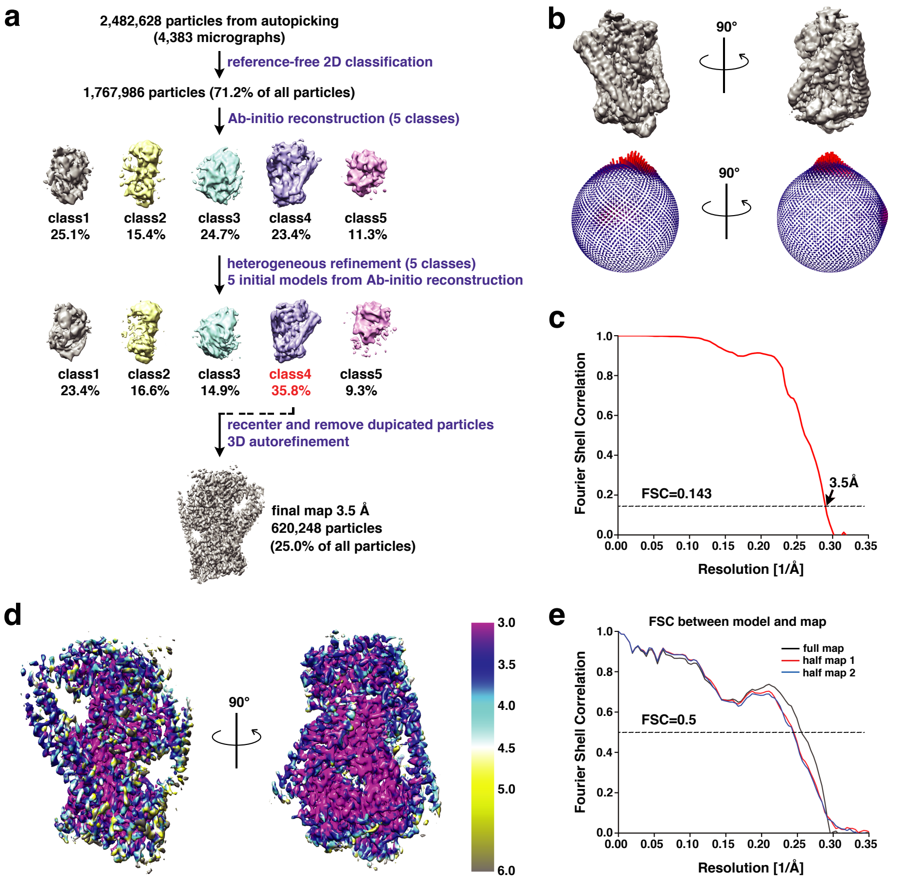

Supplementary Fig. 4. CryoEM structural determination and evaluation for the DDR' complex.

a, Data processing workflow. Please refer to Materials and Methods for more details. **b**, Angular distribution for all particles used for the final 3.5 Å map of the DDR' complex. **c**, FSC as a function of spatial frequency demonstrating the resolution for the final reconstruction of the DDR' complex. **d**, Resmap local resolution estimation. **e**, FSC coefficients as a functional of spatial frequency between model and cryoEM density maps. The generally similar appearances between the FSC curves obtained with half maps with (red) and without (blue) model refinement indicate that the refinement of the atomic coordinates did not suffer from severe over-fitting.

Supplementary Figure 5

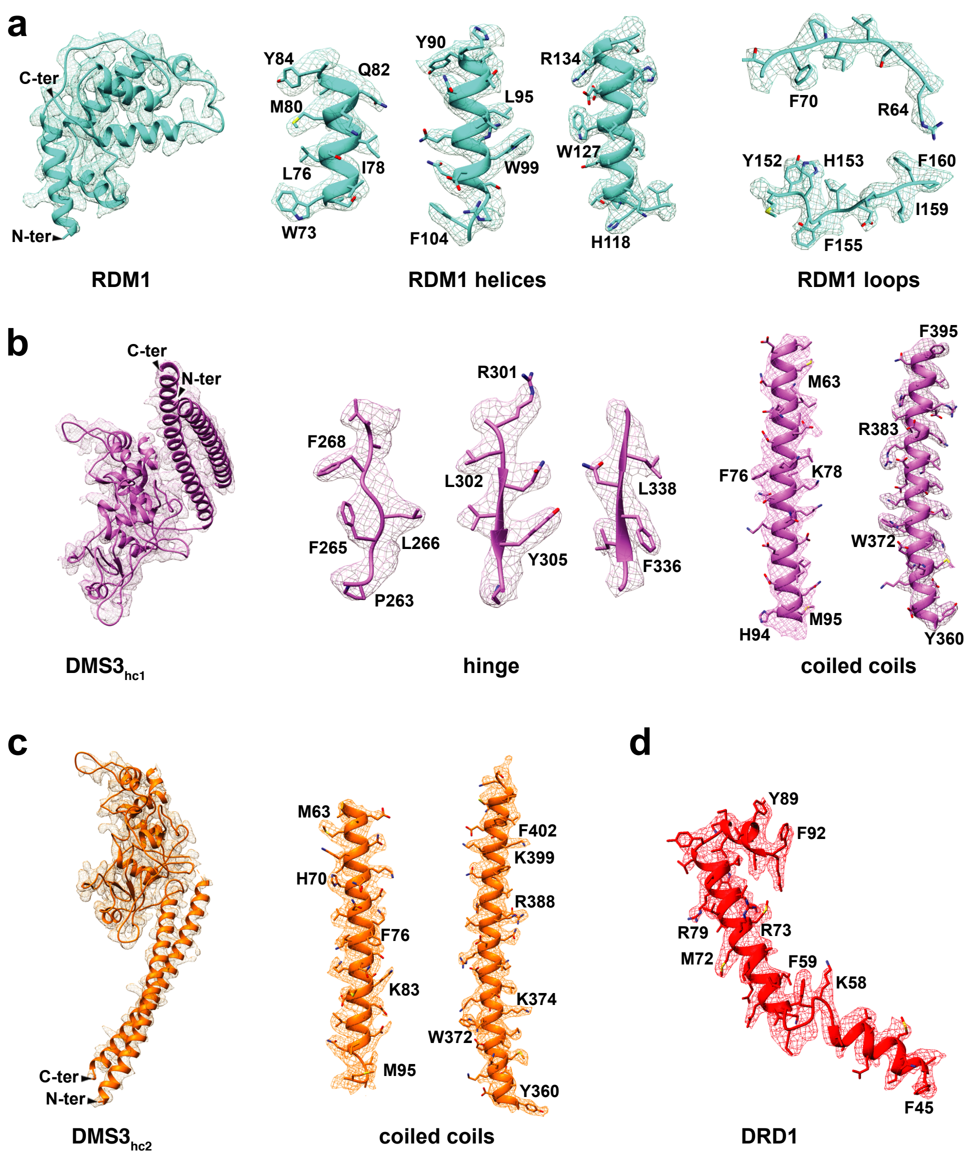

Supplementary Fig. 5. Representative cryoEM density maps of the DR and DDR' complex.

Panels (**a**, **b**) are densities for the DR complex and (**c**, **d**) are densities for the DDR' complex. The cryoEM density maps are shown for RDM1 (**a**), DMS3$_{hc1}$ (**b**), DMS3$_{hc2}$ (**c**), and DRD1 peptide (**d**).

Supplementary Figure 6

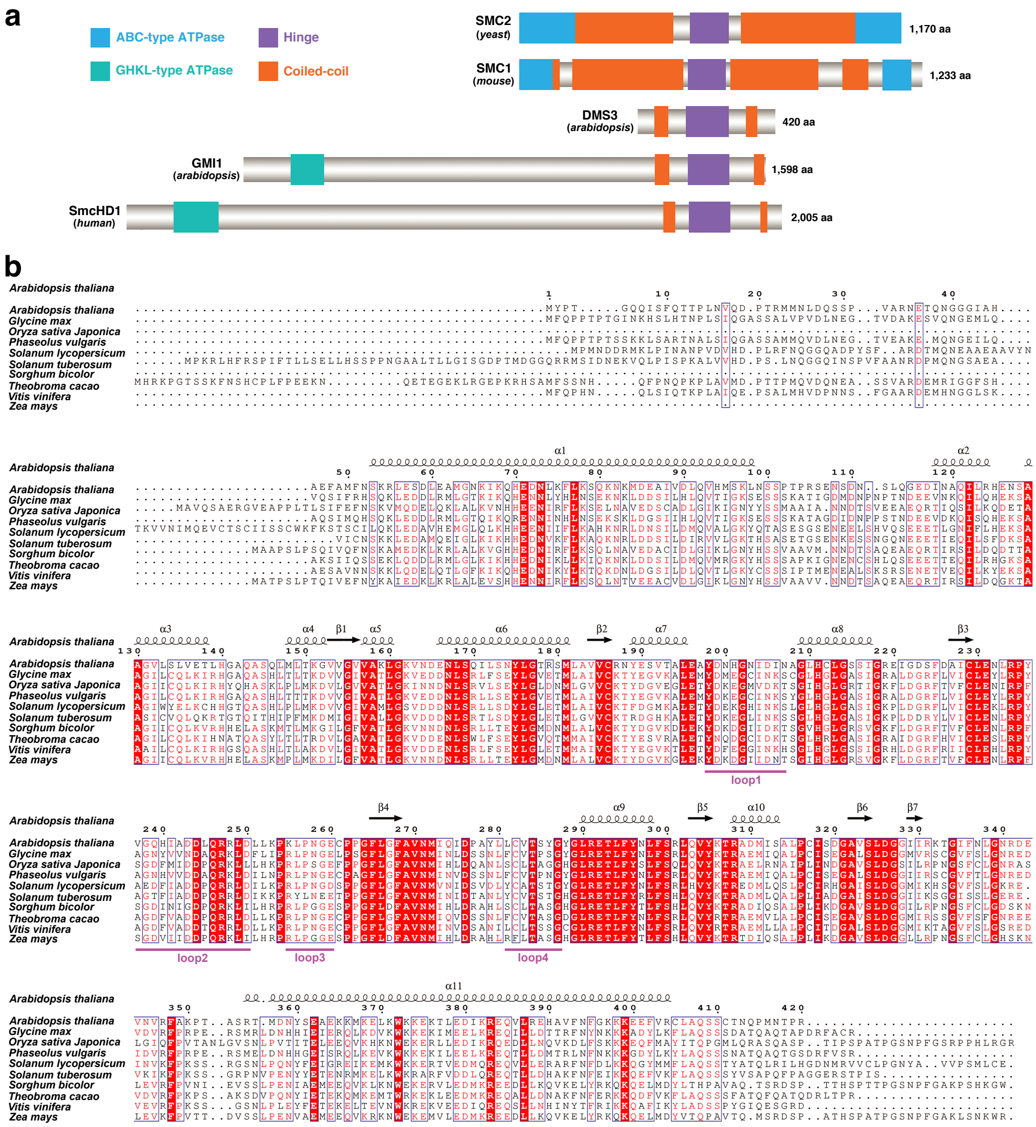

Supplementary Fig. 6. Comparison of DMS3 and other SMC hinge-containing proteins.

a, Domain structures of DMS3 and other SMC hinge-containing proteins. Protein DMS3 and GMI1 are derived from Arabidopsis thaliana. **b**, Sequence alignment of the DMS3 proteins from different species using Clustal Omega server [1] . Red background marks sequences with strict identity (100%); blue box marks sequences with more than 70% similarity; red text marks conserved amino acids.

Supplementary Figure 7

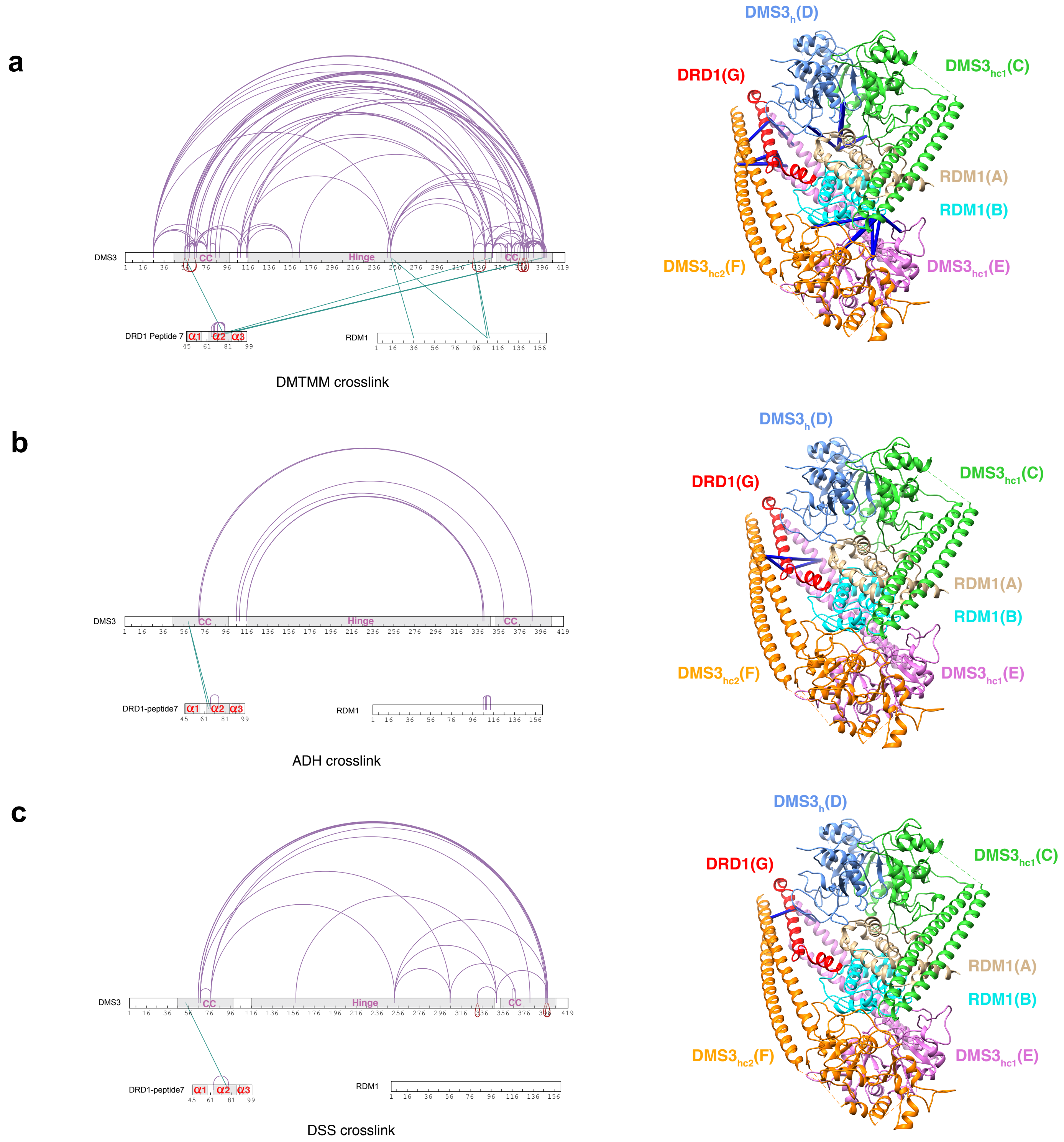

Supplementary Fig. 7. Crosslinking mass spectrometry of DDR’ complex.

Left panel of each view: schematic representations of intra- and inter- molecular crosslinks (purple and green lines, respectively) of proteins in the DDR’ complex. Visualization is performed with xiNET [2] . Known regions are grey-shaded. CC, coiled-coil. Right panel of each view: mapping inter-crosslinks shown in the corresponding left panel onto the atomic model of DDR’ (blue lines). Chain names are shown inside the parentheses. Three different types of crosslinks are shown, i.e. DMTMM or zero-length crosslink (**a**), ADH crosslink (**b**) and DSS crosslink (**c**). Zero-length links induced by DMTMM result in the highest number of intra- and inter-molecular contacts.

Supplementary Figure 8

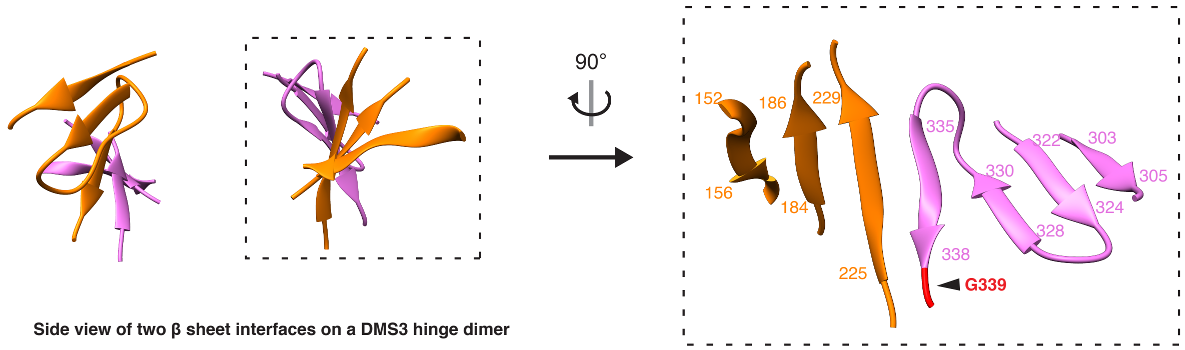

Supplementary Fig. 8. DMS3 hinge dimer interface.

Left: two reciprocal contacts of two β sheets from each DMS3 monomer (orange and pink). Right: orthogonal view of one of the contacts. Numbers mark starting and ending position of each β strand. Glycine 339 is located at the interface of the contact.

Supplementary Figure 9

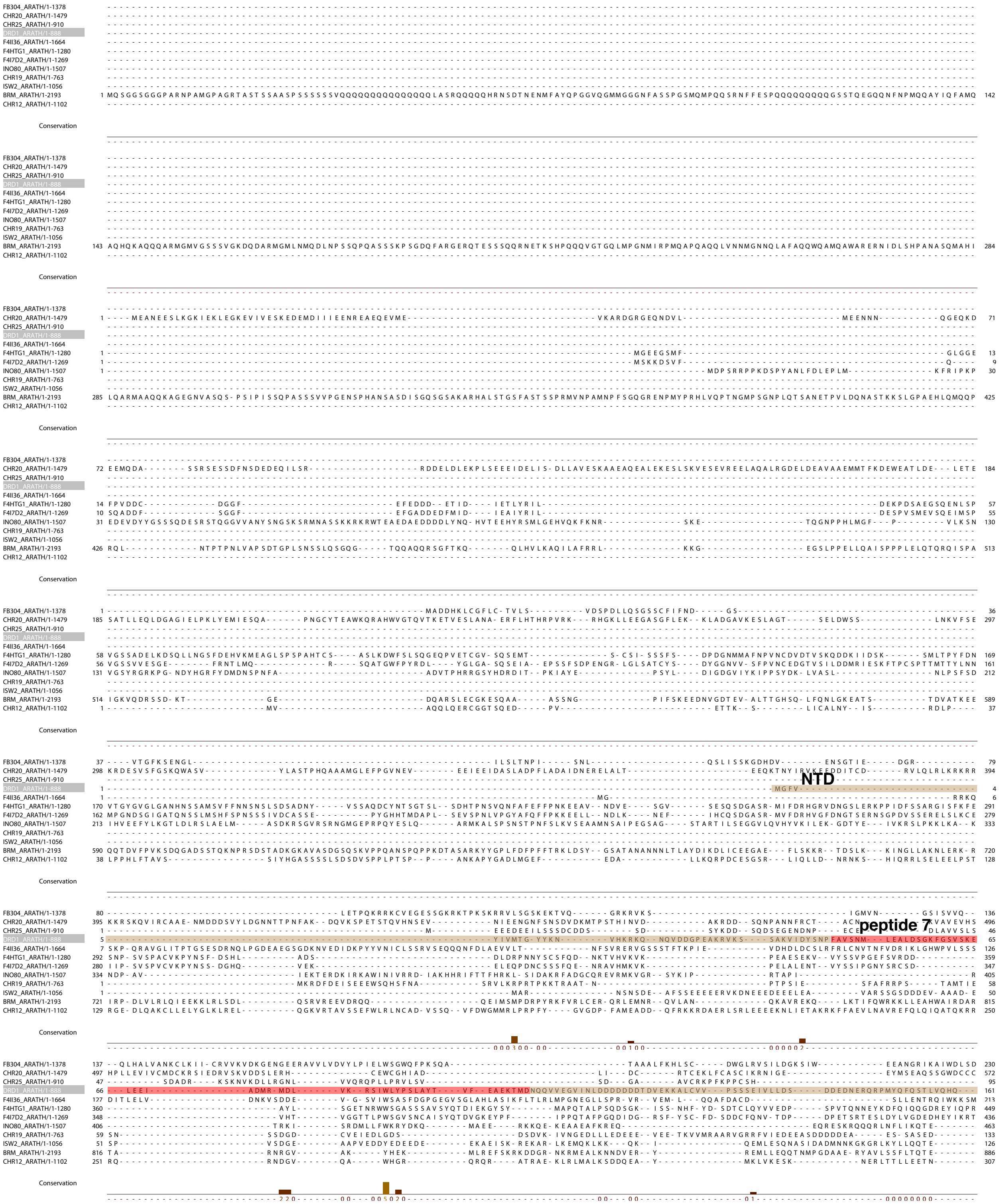

(the alignment continues next page)

Supplementary Fig. 9. Sequence comparison of Arabidopsis DRD1 and other members of SNF2 family.

Sequences of DRD1 and 11 others SNF2 family members are aligned using Clustal Omega server[1]. N-terminal domain (NTD), peptide 7, Helicase ATP-binding and Helicase C-terminal of DRD1 are highlighted with brown, red, green and blue color, respectively. Sequence identity is highlighted with shades of purple (dark purple means high identity). Sequence conservation is represented as a color-coded bar graph underneath the sequences (yellow means high conservation).

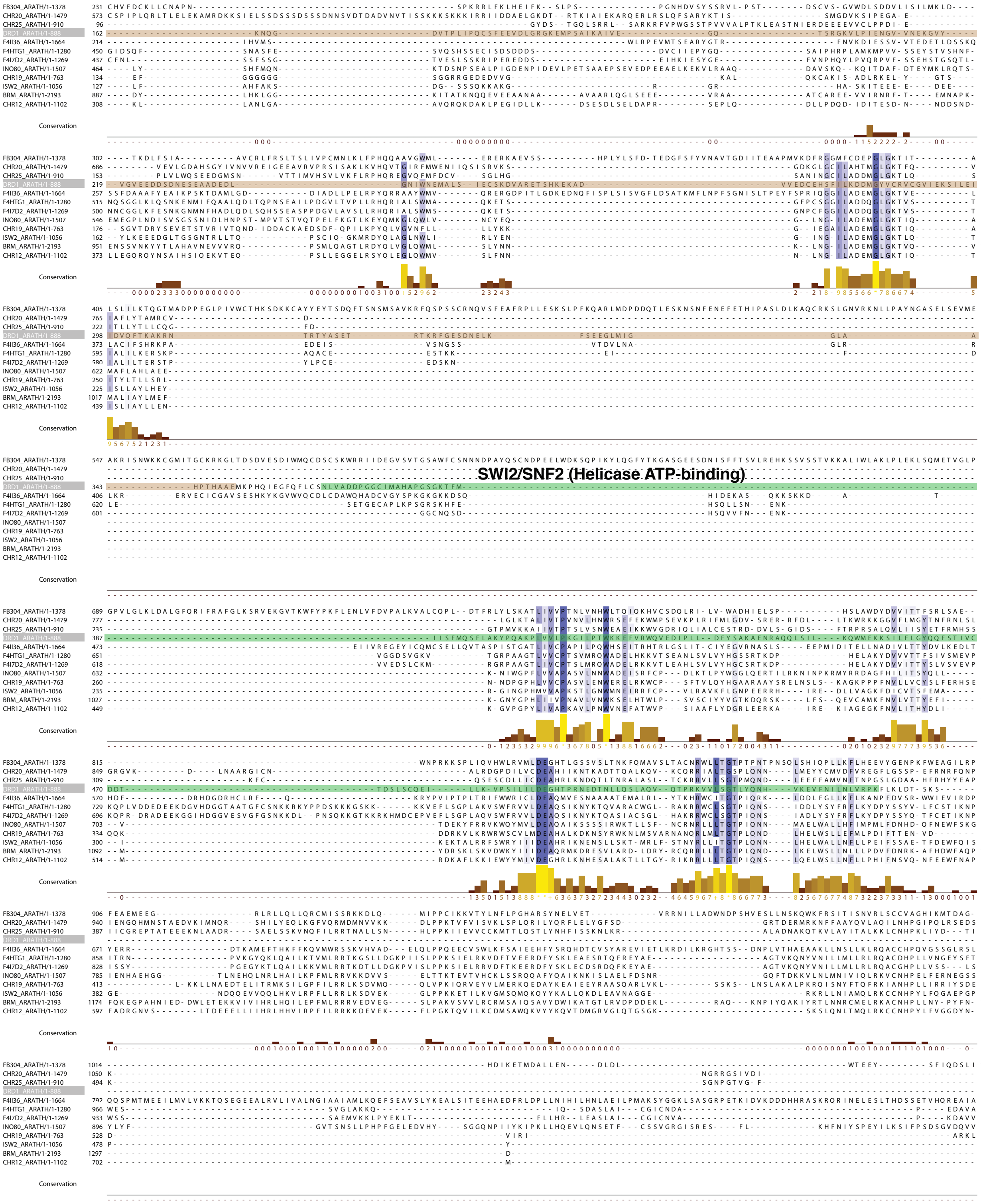

(the alignment continues next page)

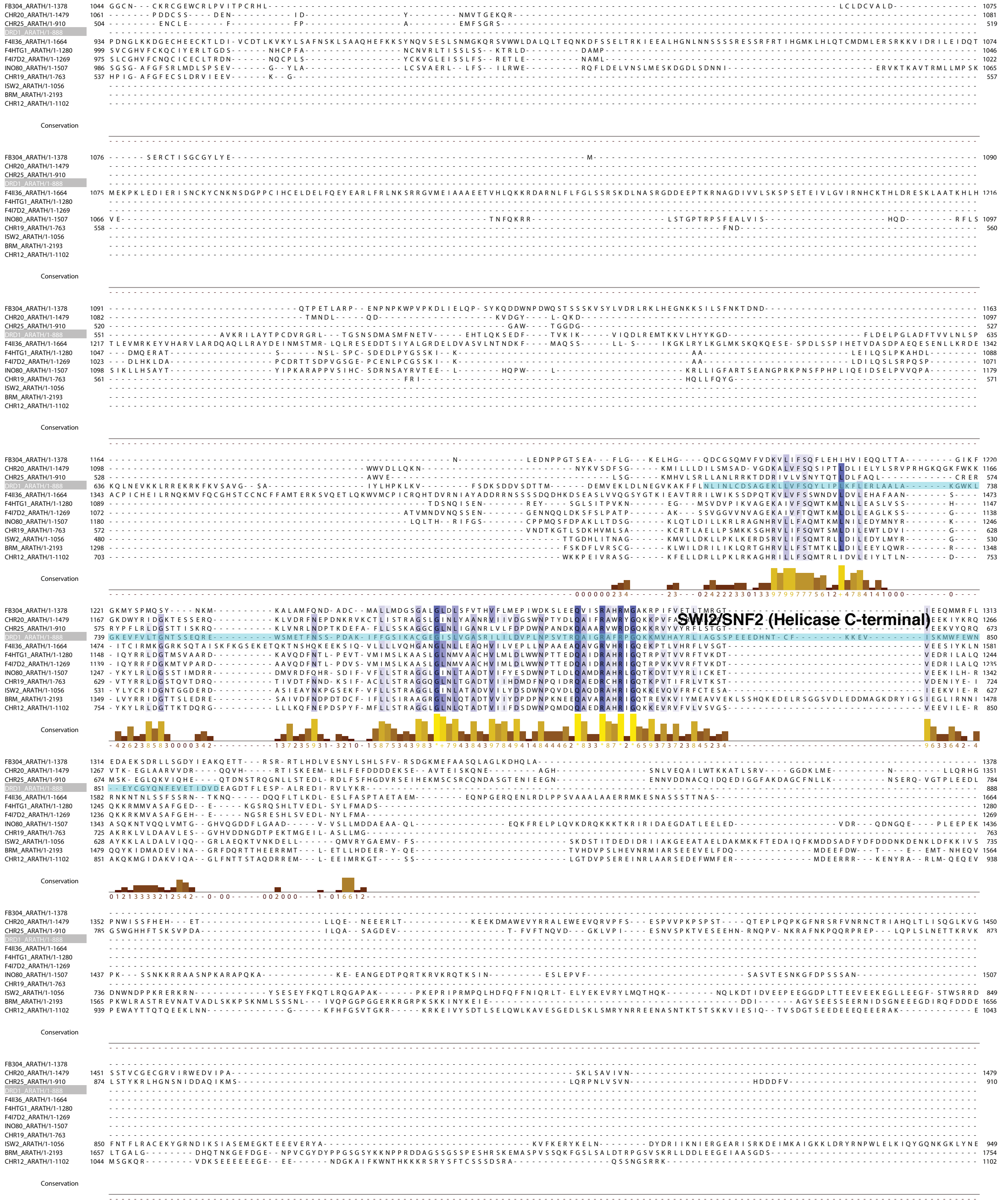

(the alignment continues next page)

```
FB304_ARATH/1-1378          ----------------------------------------------------------------------------------------------------------------------------------------------
CHR20_ARATH/1-1479          ----------------------------------------------------------------------------------------------------------------------------------------------
CHR25_ARATH/1-910           ----------------------------------------------------------------------------------------------------------------------------------------------
DRD1_ARATH/1-888            ----------------------------------------------------------------------------------------------------------------------------------------------
F4II36_ARATH/1-1664         ----------------------------------------------------------------------------------------------------------------------------------------------
F4HTG1_ARATH/1-1280         ----------------------------------------------------------------------------------------------------------------------------------------------
F4I7D2_ARATH/1-1269         ----------------------------------------------------------------------------------------------------------------------------------------------
INO80_ARATH/1-1507          ----------------------------------------------------------------------------------------------------------------------------------------------
CHR19_ARATH/1-763           ----------------------------------------------------------------------------------------------------------------------------------------------
ISW2_ARATH/1-1056     950   ECDRFMICMIHKLGYGNWDELKAAFRTSSVFRFDWFVKSRTSQELARRCDTLIRLIEKENQEFDERERQARKEKKLAKSATPSKRPLGRQASE---------SPSSTK--------------------KRKHLS--------   1054
BRM_ARATH/1-2193      1755  -------HIDLQRSGSWAHDRDEGDEEQVLQPT--IKRK----------RSIRLRP-----------R--QTAERVDGSEMPAAQPLQVDRSYRSKLRTVVDSHSSRQDQSDSSSRLRSVPAKKVASTSKLHVSSPKSGRLN   1864
CHR12_ARATH/1-1102          ----------------------------------------------------------------------------------------------------------------------------------------------

Conservation
                            ----------------------------------------------------------------------------------------------------------------------------------------------

FB304_ARATH/1-1378          ----------------------------------------------------------------------------------------------------------------------------------------------
CHR20_ARATH/1-1479          ----------------------------------------------------------------------------------------------------------------------------------------------
CHR25_ARATH/1-910           ----------------------------------------------------------------------------------------------------------------------------------------------
DRD1_ARATH/1-888            ----------------------------------------------------------------------------------------------------------------------------------------------
F4II36_ARATH/1-1664         ----------------------------------------------------------------------------------------------------------------------------------------------
F4HTG1_ARATH/1-1280         ----------------------------------------------------------------------------------------------------------------------------------------------
F4I7D2_ARATH/1-1269         ----------------------------------------------------------------------------------------------------------------------------------------------
INO80_ARATH/1-1507          ----------------------------------------------------------------------------------------------------------------------------------------------
CHR19_ARATH/1-763           ----------------------------------------------------------------------------------------------------------------------------------------------
ISW2_ARATH/1-1056     1055  ----MR--------------------------------------------------------------------------------------------------------------------------------------   1056
BRM_ARATH/1-2193      1865  ATQLTVEDNAEASRETWDGTSPISSSNAGARMSHIIQKRCKIVISKLQRRIDKEGQQIVPMLTNLWKRIQNGYAAGGVNNLLELREIDHRVERLEYAGVMELASDVQLMLRGAMQFYGFSHEVRSEAKKVHNLFFDLLKMSF   2006
CHR12_ARATH/1-1102          ----------------------------------------------------------------------------------------------------------------------------------------------

Conservation
                            ----------------------------------------------------------------------------------------------------------------------------------------------

FB304_ARATH/1-1378          ----------------------------------------------------------------------------------------------------------------------------------------------
CHR20_ARATH/1-1479          ----------------------------------------------------------------------------------------------------------------------------------------------
CHR25_ARATH/1-910           ----------------------------------------------------------------------------------------------------------------------------------------------
DRD1_ARATH/1-888            ----------------------------------------------------------------------------------------------------------------------------------------------
F4II36_ARATH/1-1664         ----------------------------------------------------------------------------------------------------------------------------------------------
F4HTG1_ARATH/1-1280         ----------------------------------------------------------------------------------------------------------------------------------------------
F4I7D2_ARATH/1-1269         ----------------------------------------------------------------------------------------------------------------------------------------------
INO80_ARATH/1-1507          ----------------------------------------------------------------------------------------------------------------------------------------------
CHR19_ARATH/1-763           ----------------------------------------------------------------------------------------------------------------------------------------------
ISW2_ARATH/1-1056           ----------------------------------------------------------------------------------------------------------------------------------------------
BRM_ARATH/1-2193      2007  PDTDFREARNALSFSGSAPTLVSTPTPRGAGISQGKRQKLVNEPETEPSSPQRSQQRENSRIRVQIPQKETKLGGTTSHTDESPILAHPGELVICKKKRKDREKSGPKTRTGGSSSPVSPPPAMIGRGLRSPVSGGVPRETR   2148
CHR12_ARATH/1-1102          ----------------------------------------------------------------------------------------------------------------------------------------------

Conservation
                            ----------------------------------------------------------------------------------------------------------------------------------------------

FB304_ARATH/1-1378          ---------------------------------------------
CHR20_ARATH/1-1479          ---------------------------------------------
CHR25_ARATH/1-910           ---------------------------------------------
DRD1_ARATH/1-888            ---------------------------------------------
F4II36_ARATH/1-1664         ---------------------------------------------
F4HTG1_ARATH/1-1280         ---------------------------------------------
F4I7D2_ARATH/1-1269         ---------------------------------------------
INO80_ARATH/1-1507          ---------------------------------------------
CHR19_ARATH/1-763           ---------------------------------------------
ISW2_ARATH/1-1056           ---------------------------------------------
BRM_ARATH/1-2193      2149  LAQQQRWPNQPTHPNNSGAAGDSVGWANPVKRLRTDSGKRRPSHL   2193
CHR12_ARATH/1-1102          ---------------------------------------------

Conservation
                            ---------------------------------------------
```

Supplementary Figure 10

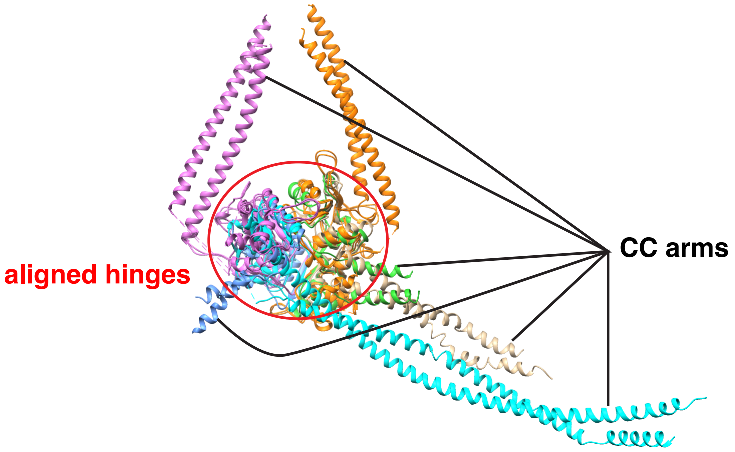

superimposition of the hinge domains of DMS3 and SMC proteins

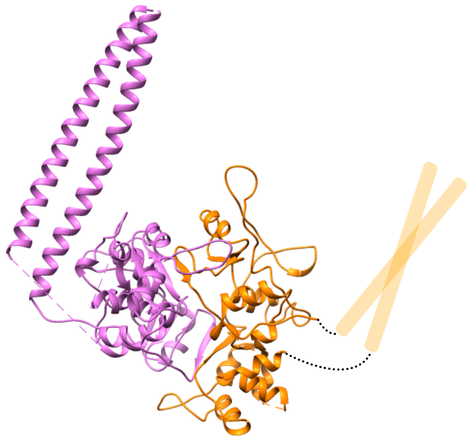

Arabidopsis thaliana **DMS3 hinge with "open" CC arms DDR'**

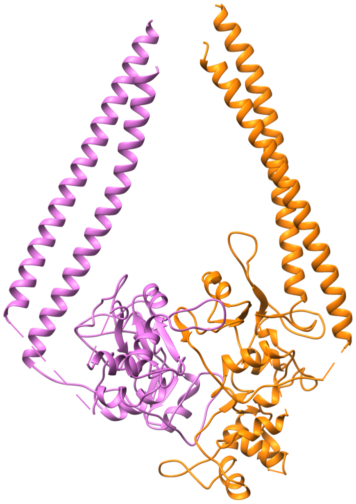

Arabidopsis thaliana **DMS3 hinge with "closed" CC arms DDR'**

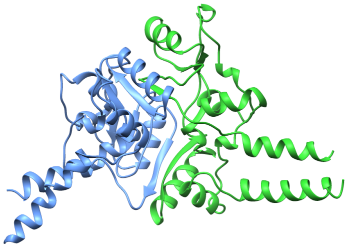

T. maritima **Smc hinge with "open" CC arms PDB ID: 1GXL**

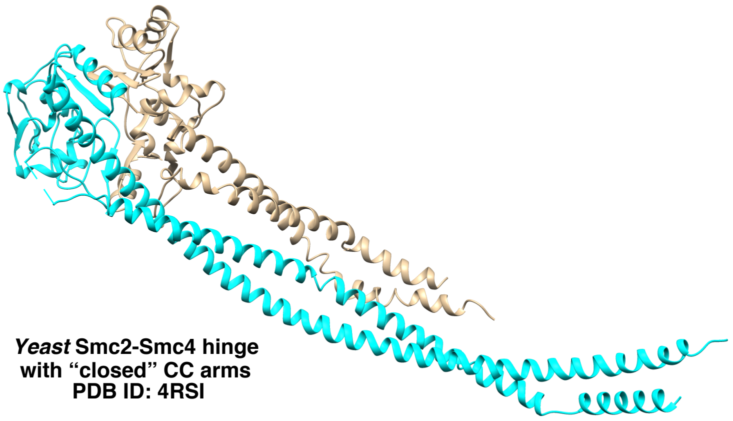

Yeast **Smc2-Smc4 hinge with "closed" CC arms PDB ID: 4RSI**

Supplementary Fig. 10. Structural comparison of DMS3 and SMC proteins

Top panel: structural superimposition of the hinge domains of DMS3 and SMC proteins revealing the different orientation of their CC arms; middle panels: the CC arms in open (left) and closed (right) conformations of DMS3; bottom panels: the CC arms in open (left) and closed (right) conformations of SMC proteins.

Supplementary Figure 11

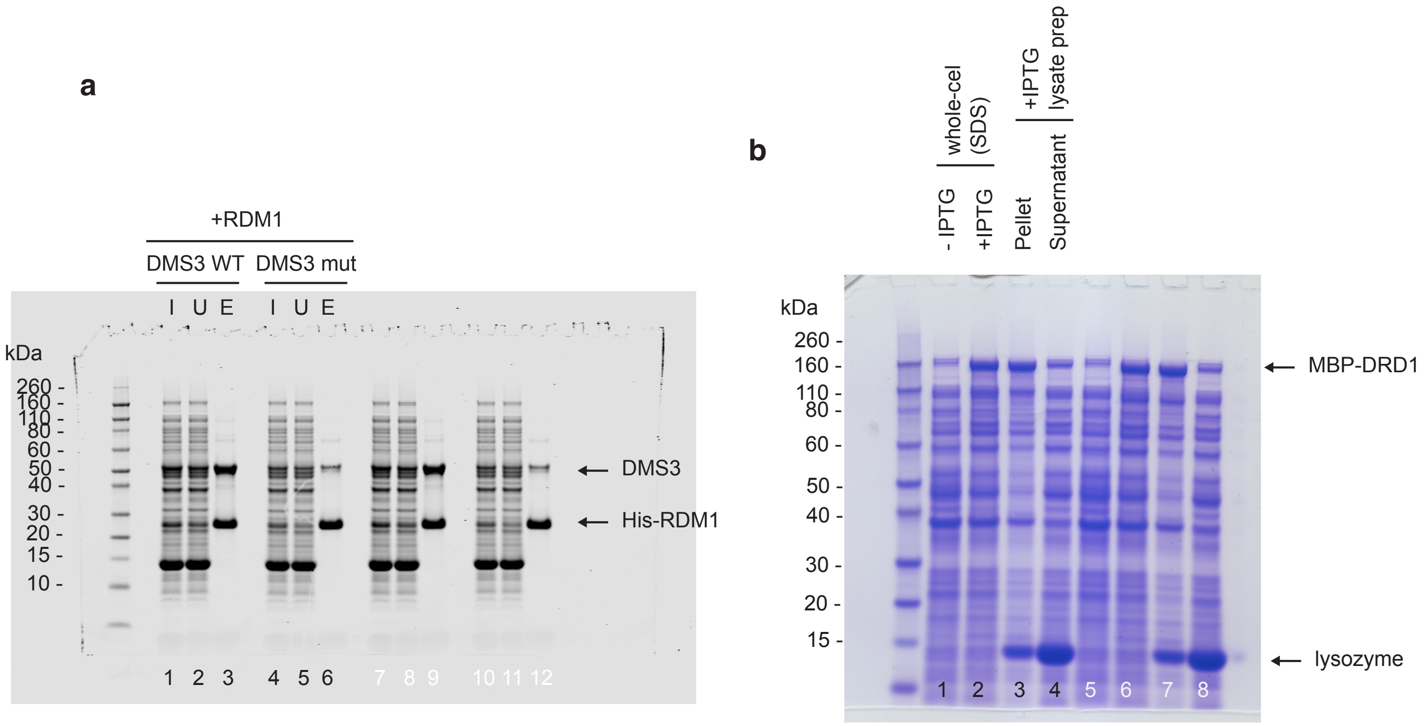

Supplementary Fig. 11. Uncropped scanned gel images from Fig. 2c and Supplementary Fig. 1d.

a, Uncropped scanned gel image of Fig. 2c. The image is derived from combining scans from both 700-nm and 800-nm channels (Odyssey). Lanes 1-6 are shown in Fig. 2c. A duplicate experiment is from lanes 7-12. **b**, Uncropped scanned gel image of Supplementary Fig. 1d. The image is derived from a scan on a white light scanner. Lanes 5-8 are not relevant to this study.

Supplementary References

1. Sievers, F. *et al*. Fast, scalable generation of high-quality protein multiple sequence alignments using Clustal Omega. *Mol. Syst. Biol.* **7**, 539 (2011).

2. Combe, C. W., Fischer, L. & Rappsilber, J. xiNET: cross-link network maps with residue resolution. *Mol. Cell Proteomics* **14**, 1137–1147 (2015).
