# Supplementary material for: CryoEM structures of Arabidopsis DDR complexes involved in RNA-directed DNA methylation
Source: Nat Commun. 2019 Sep 2;10:3916. doi: 10.1038/s41467-019-11759-9 (PMC6718625; doi:10.1038/s41467-019-11759-9)
Supplement: Supplementary file 2 — Description of Additional Supplementary Files [file 41467_2019_11759_MOESM2_ESM.pdf]

Description of Additional Supplementary Files

File Name: Supplementary Data 1

Description: Identification of crosslinked peptides derived from the DDR' complex treated with various crosslinkers.
