# Supplementary material for: CryoEM structures of Arabidopsis DDR complexes involved in RNA-directed DNA methylation
Source: Nat Commun. 2019 Sep 2;10:3916. doi: 10.1038/s41467-019-11759-9 (PMC6718625; doi:10.1038/s41467-019-11759-9)
Supplement: Supplementary file 4 — Reporting Summary [file 41467_2019_11759_MOESM4_ESM.pdf]

nature research

Corresponding author(s): Steve E Jacobsen and Z Hong Zhou

Last updated by author(s): Jul 15, 2019

Reporting Summary

Nature Research wishes to improve the reproducibility of the work that we publish. This form provides structure for consistency and transparency in reporting. For further information on Nature Research policies, see Authors & Referees and the Editorial Policy Checklist.

Statistics

For all statistical analyses, confirm that the following items are present in the figure legend, table legend, main text, or Methods section.

n/a	Confirmed	
☐	☒	The exact sample size (*n*) for each experimental group/condition, given as a discrete number and unit of measurement
☐	☒	A statement on whether measurements were taken from distinct samples or whether the same sample was measured repeatedly
☒	☐	The statistical test(s) used AND whether they are one- or two-sided<br>*Only common tests should be described solely by name; describe more complex techniques in the Methods section.*
☒	☐	A description of all covariates tested
☒	☐	A description of any assumptions or corrections, such as tests of normality and adjustment for multiple comparisons
☒	☐	A full description of the statistical parameters including central tendency (e.g. means) or other basic estimates (e.g. regression coefficient) AND variation (e.g. standard deviation) or associated estimates of uncertainty (e.g. confidence intervals)
☒	☐	For null hypothesis testing, the test statistic (e.g. *F*, *t*, *r*) with confidence intervals, effect sizes, degrees of freedom and *P* value noted<br>*Give P values as exact values whenever suitable.*
☐	☒	For Bayesian analysis, information on the choice of priors and Markov chain Monte Carlo settings
☒	☐	For hierarchical and complex designs, identification of the appropriate level for tests and full reporting of outcomes
☒	☐	Estimates of effect sizes (e.g. Cohen's *d*, Pearson's *r*), indicating how they were calculated

Our web collection on statistics for biologists contains articles on many of the points above.

Software and code

Policy information about availability of computer code

Data collection	Specified or referenced in Materials and Methods section
Data analysis	Specified or referenced in Materials and Methods section

For manuscripts utilizing custom algorithms or software that are central to the research but not yet described in published literature, software must be made available to editors/reviewers. We strongly encourage code deposition in a community repository (e.g. GitHub). See the Nature Research guidelines for submitting code & software for further information.

Data

Policy information about availability of data

All manuscripts must include a data availability statement. This statement should provide the following information, where applicable:

- Accession codes, unique identifiers, or web links for publicly available datasets
- A list of figures that have associated raw data
- A description of any restrictions on data availability

ChIP-seq, WGBS, EM, IP-MS and XL-MS data are deposited in publicly available database, with accession numbers listed in the manuscript under "Data Availability" section.

Field-specific reporting

Please select the one below that is the best fit for your research. If you are not sure, read the appropriate sections before making your selection.

☒ Life sciences ☐ Behavioural & social sciences ☐ Ecological, evolutionary & environmental sciences

For a reference copy of the document with all sections, see nature.com/documents/nr-reporting-summary-flat.pdf

Life sciences study design

All studies must disclose on these points even when the disclosure is negative.

Sample size	For cryoEM analysis, 3D reconstructions were calculated from thousands of particle images. Resolutions of core regions are measured by the FSCResmap program to be ~3.5-3.6 angstroms, with visible densities for different amino acid residue side chains.
Data exclusions	For cryoEM analysis, about 80% of the particle images were excluded after the rounds of 2D and 3D classification. These particles were regarded as having bad quality and including them in the reconstruction would dampen the high resolution structural information. It is well established principle in cryoEM that excluding these particles would not affect the structure obtained.<br>For functional studies, no data were excluded from the analyses.
Replication	Yeast 2- and 3- hybrids, IP-MS, His tag pulldown and XL-MS were successfully replicated. Multiple batches of DR and DDR' complexes were reproducibly purified and used for EM data collection. RDM1, DMS3 and DRD1 ChIPs were performed once. WGBS was performed once using two independent transgenic lines and controls.
Randomization	Randomization was not relevant, hence, not applied to these experiments.
Blinding	Blinding was not relevant, hence, not applied to these experiments.

Reporting for specific materials, systems and methods

We require information from authors about some types of materials, experimental systems and methods used in many studies. Here, indicate whether each material, system or method listed is relevant to your study. If you are not sure if a list item applies to your research, read the appropriate section before selecting a response.

Materials & experimental systems

n/a	Involved in the study
☐	☒ Antibodies
☒	☐ Eukaryotic cell lines
☒	☐ Palaeontology
☒	☐ Animals and other organisms
☒	☐ Human research participants
☒	☐ Clinical data

Methods

n/a	Involved in the study
☐	☒ ChIP-seq
☒	☐ Flow cytometry
☒	☐ MRI-based neuroimaging

Antibodies

Antibodies used	Anti-Flag M2 (Sigma, F1804; commercially available)
Validation	Anti-Flag M2 has been widely used as an epitope tag (see manufacturers' website for information).

ChIP-seq

Data deposition

☒ Confirm that both raw and final processed data have been deposited in a public database such as GEO.

☒ Confirm that you have deposited or provided access to graph files (e.g. BED files) for the called peaks.

Data access links<br>*May remain private before publication.*	https://www.ncbi.nlm.nih.gov/geo/query/acc.cgi?acc=GSE130290
Files in database submission	DRD1_FLAG.fastq.gz<br>DMS3_FLAG.fastq.gz<br>RDM1_FLAG.fastq.gz<br>Col0.fastq.gz<br>log2ratioDRD1overCol0.bw<br>log2ratioDMS3overCol0.bw<br>log2ratioRDM1overCol0.bw<br>PolV_peaks_q0.05_FC2.summit.bed
Genome browser session<br>(e.g. UCSC)	Tracks displaying DRD1, DMS3 and RDM1 ChIP-seq signal over Col0 ChIP control can be found as Supplementary files at https://www.ncbi.nlm.nih.gov/geo/query/acc.cgi?acc=GSE130290

Methodology

Replicates	ChIP-seq for each DDR component was performed once.
Sequencing depth	Col0_ChIPseq_rep1: 29830446 reads total, 24855412 uniquely mapped, 50bp, single end.<br>DRD1_ChIPseq_rep2: 26439435 reads total, 21566096 uniquely mapped, 50bp, single end.<br>DMS3_ChIPseq_rep2: 27787594 reads total, 23668149 uniquely mapped, 50bp, single end.<br>RDM1_ChIPseq_rep2: 24735665 reads total, 20203109 uniquely mapped, 50bp, single end.
Antibodies	anti-Flag M2 antibody (Sigma, F1804)
Peak calling parameters	Pol V peaks were called comparing Pol V antibody ChIP against no antibody control ChIP described in Liu et al., Nature Plants 2018, using MACS2 with q-value 0.05 (v 2.1.1). Only peaks with more then 2FC were used.
Data quality	The antibody used for the ChIPs is widely used for ChIP experiments. The correct size of chromatin and libraries was checked in a gel. Only raw reads passing the QC were used for alignment. Tracks for each ChIP were generated and inspected in order to check the quality of the data.
Software	See Materials and Methods section
